# Increased levels of microRNA‐320 in blood serum and plasma is associated with imminent and advanced lung cancer

**DOI:** 10.1002/1878-0261.13336

**Published:** 2022-11-27

**Authors:** Therese Haugdahl Nøst, Anne Heidi Skogholt, Ilona Urbarova, Robin Mjelle, Erna‐Elise Paulsen, Tom Dønnem, Sigve Andersen, Maria Markaki, Oluf Dimitri Røe, Mikael Johansson, Mattias Johansson, Bjørn Henning Grønberg, Torkjel Manning Sandanger, Pål Sætrom

**Affiliations:** ^1^ Department of Community Medicine, Faculty of Health Sciences UiT The Arctic University of Norway Tromsø Norway; ^2^ Department of Public Health and Nursing, K.G. Jebsen Center for Genetic Epidemiology NTNU – Norwegian University of Science and Technology Trondheim Norway; ^3^ Department of Clinical and Molecular Medicine NTNU – Norwegian University of Science and Technology Trondheim Norway; ^4^ Bioinformatics Core Facility NTNU – Norwegian University of Science and Technology Trondheim Norway; ^5^ Department of Clinical Medicine, Faculty of Health Sciences UiT The Arctic University of Norway Tromsø Norway; ^6^ Department of Pulmonology University Hospital of North Norway Tromsø Norway; ^7^ Department of Oncology University Hospital of North Norway Tromsø Norway; ^8^ Institute of Computer Science Crete Greece; ^9^ Cancer Clinic, Levanger Hospital Nord‐Trøndelag Health Trust Levanger Norway; ^10^ Department of Radiation Sciences, Oncology Umeå University Sweden; ^11^ International Agency for Research on Cancer Lyon France; ^12^ Department of Oncology, St. Olavs Hospital Trondheim University Hospital Norway; ^13^ Department of Computer Science Norwegian University of Science and Technology Trondheim Norway

**Keywords:** diagnostic markers, lung cancer, MiRNA, peripheral blood, pre‐diagnostic markers

## Abstract

Lung cancer (LC) incidence is increasing globally and altered levels of microRNAs (miRNAs) in blood may contribute to identification of individuals with LC. We identified miRNAs differentially expressed in peripheral blood at LC diagnosis and evaluated, in pre‐diagnostic blood specimens, how long before diagnosis expression changes in such candidate miRNAs could be detected. We identified upregulated candidate miRNAs in plasma specimens from a hospital‐based study sample of 128 patients with confirmed LC and 62 individuals with suspected but confirmed negative LC (FalsePos). We then evaluated the expression of candidate miRNAs in pre‐diagnostic plasma or serum specimens of 360 future LC cases and 375 matched controls. There were 1663 miRNAs detected in diagnostic specimens, nine of which met our criteria for candidate miRNAs. Higher expression of three candidates, miR‐320b, 320c, and 320d, was associated with poor survival, independent of LC stage and subtype. Moreover, miR‐320c and miR‐320d expression was higher in pre‐diagnostic specimens collected within 2 years of LC diagnosis. Our results indicated that elevated levels of miR‐320c and miR‐320d may be early indications of imminent and advanced LC.

## Graphical abstract

We applied a novel approach of identifying early markers of lung cancer using both diagnostic and pre‐diagnostic blood specimens. We detected elevated levels of miR‐320c and miR‐320d in blood up to 2 years prior to the lung cancer diagnosis. These two miRNAs were identified as the most promising markers and may be early indications of imminent and advanced lung cancer.

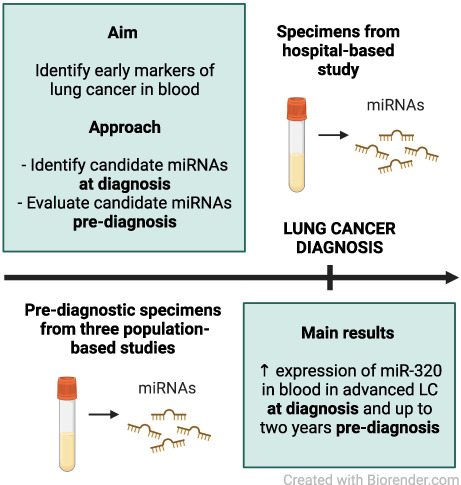

**Abbreviations**
AUC: area under the curve
FalsePos: individuals with suspected LC but negative LC diagnostic evaluation
FC: fold change
FDR: false‐discovery rates
HUNT: the Trøndelag Health Study
LC: lung cancer
miRNA: microRNA
NLCB: the Norwegian Lung Cancer Biobank
NOWAC: the Norwegian Women and Cancer Study
NSCLC: non‐small cell lung cancer
NSHDS: the Northern Sweden Health and Disease Study
OR: odds ratio
ROC: receiver operating characteristic curves
RPM: reads per million
SCLC: small cell lung cancer

## 1 Introduction

Lung cancer (LC) is the leading cause of cancer death worldwide, with more than 1.8 million deaths in 2020 [1]. Lung cancer is a heterogeneous disease, and the most common histological subtypes are non‐small cell LC [NSCLC; with the dominating entities adenocarcinoma (AD) and squamous cell carcinoma (SQ)] and small cell LC (SCLC). A high proportion of LC cases is diagnosed with advanced disease [2, 3], especially those with SCLC [4]. Survival is strongly related to stage at LC diagnosis [3, 5]; therefore, improved methods for the identification of individuals at high risk who should undergo regular screening, and for the diagnosis of LC at an early stage when curative treatment can be offered, are needed to reduce LC mortality. Although population‐based LC screening programs have been reported to contribute to increased overall survival [6], they are resource‐intensive and confer a considerable risk of overdiagnosis and overtreatment [7], thus improved accuracy of screening tests is also needed.

The discovery of pre‐diagnostic blood markers is key to identifying high‐risk individuals before the manifestation of advanced LC. Blood‐based tests are minimally invasive and accessible, and could serve to identify high‐risk individuals who may benefit from LC screening. Indeed, the identification of diagnostic and prognostic markers for personalized treatment and follow‐up of LC patients with different histological subtypes is the focus of considerable research efforts worldwide [8]. Currently, there are no biomarkers for LC successfully implemented in large‐scale clinical or screening setting for early detection but there are promising candidates. Circulating microRNAs (miRNAs) are potential biomarkers for several cancers [9, 10] and have shown promise as diagnostic markers, prognostic markers, and treatment prediction markers in LC [11, 12, 13, 14, 15, 16, 17, 18, 19]. Indeed, miRNAs was included in the Multicentric Italian Lung Detection trial, which recently reported better risk stratification when blood markers were used in addition to a low‐dose computed tomography [6, 13].

There is substantial variation across studies in the miRNAs reported to be associated with LC, and only a few studies with modest sample sizes have investigated pre‐diagnostic blood specimens [20, 21, 22, 23]. It is therefore unclear to what extent miRNAs that are associated with LC at diagnosis are also associated with LC prior to diagnosis. Moreover, if such a pre‐diagnostic association exists, it is unclear how long before clinical diagnosis changes in expression of these miRNAs can be detected.

To address these issues, we identified differentially expressed candidate miRNAs in diagnostic blood specimens of individuals with LC. Further, we evaluated their presence in pre‐diagnostic blood specimens of individuals with LC, how long before diagnosis candidate miRNAs could be detected, and determined their diagnostic and predictive value for LC.

## 2 Materials and methods

### 2.1 Study sample

This work included blood specimens together with medical records, questionnaire, or health registry data from four separate cohort studies: one hospital‐based study and three pre‐diagnostic, population‐based studies. Specifically, we included blood specimens collected: (a) at the time of diagnosis, in the Norwegian Lung Cancer Biobank (NLCB) during diagnostic workup at a hospital and (b) blood specimens collected pre‐diagnostically in three prospective studies: the Norwegian Women and Cancer Study (NOWAC), the Northern Sweden Health and Disease Study (NSHDS), and the Trøndelag Health Study (HUNT). We classified LC cases [International Classification of Diseases (ICD‐10)] topography codes (C33–C34) as early‐, middle‐, and late‐stage based on information from medical records for data in the NLCB and NSHDS studies (TNM status), and the national cancer registry for data in the NOWAC and HUNT studies (classification by the registry, see Table S1). The aim was to construct staging information that could be harmonized across data sources, and included early‐stage (local disease), middle‐stage (regional disease or spread), and late‐stage cases (advanced or systemic disease that had spread to the whole body). All participants have given written informed consent to the respective cohorts and the studies have been approved by the respective Regional Committees for Medical and Health Research Ethics in Norway and Sweden. The research has been conducted according to the principles expressed in the Declaration of Helsinki.

#### 2.1.1 Hospital‐based study sample for the identification of candidate microRNAs in diagnostic specimens

The hospital‐based study sample was based on the NLCB, a disease‐specific biobank with ongoing recruitment that was established in 2005 at St. Olavs Hospital and Norwegian University of Science and Technology (NTNU) in Trondheim, Norway. The recruited participants included individuals that were symptomatic of LC and were undergoing diagnostic evaluation for LC, mainly based on findings on imaging (CT scans). This study sample comprised patients who were positively diagnosed with LC, of any histological subtype and stage, and individuals for whom the diagnostic work up concluded did not have LC. The latter group was included as a control group and considered ‘false positives’ (hereafter referred to as FalsePos). Case ascertainment was ensured by reviewing information from medical records. During diagnostic workup, specimens from lung and blood were collected together with phenotype data. Serum specimens were collected at recruitment into the study during diagnostic workup and prior to any treatment. Phenotype data were collected from questionnaires and electronic health records from the hospital. Plasma, serum, and RNA stabilizing tubes (PAXgene) were collected and stored at −80 °C. In total, 87 individuals were classified as FalsePos and among these, 25 had a previous cancer diagnosis and were excluded from the statistical analyses.

#### 2.1.2 Prospective study sample for the evaluation of candidate microRNAs in pre‐diagnostic specimens

The prospective study sample included individuals from three prospective studies: NOWAC, NSHDS, and HUNT. Lung cancer cases were identified using linkages to national cancer registries in Norway and Sweden, and cases with blood specimens collected before LC diagnosis were included. One matched control was identified for each case within the respective studies. The matching criteria between cases and controls, time to diagnosis, and follow‐up time differed somewhat across the three prospective studies. Common matching criteria included age and sex, and the common interval for time to diagnosis was 0.11–5 years, whereas the year of the latest LC diagnosis varied from 2005 (HUNT) to 2017 (NSHDS).

NOWAC is a nationally representative cohort study initiated in 1991 [24] established at UiT The Arctic University of Norway. Women aged 30–70 years were randomly selected from the National Registry and invited to participate in the study. Participants filled out a questionnaire at recruitment and have been followed up with up to three questionnaires since then. The questionnaires have covered self‐reported anthropometry and lifestyle variables, including detailed information on past and concurrent smoking. We conducted a case–control study nested within the NOWAC study among those participants who had donated a blood specimen in 2003–2006 (*N* = 48 941). At the time of blood donation, the participants also filled out a one‐page questionnaire covering information about recent and current smoking habits. Plasma specimens were collected and stored at −80 °C. Through linkage with the Cancer Registry of Norway we identified 134 participants who had been diagnosed with LC after they donated a blood specimen. For each case, one cancer‐free control was randomly drawn from NOWAC participants with available blood specimens and matched on birth year and blood specimen collection batch.

The HUNT Study is a population‐based health survey established at NTNU [25]. All inhabitants aged 20 years or older in the northern area of Trøndelag have been invited to four surveys: HUNT1‐4. More than 120 000 individuals have participated and responded to questionnaires and donated blood specimens. A nested case–control study was designed within participants in the HUNT2 survey, including 120 incident cases who developed LC after donating blood specimens in 1995–1997 and 120 controls matched on sex, age, pack‐years, and years since quitting. Incident LC cases were identified by linkage with the Cancer Registry of Norway. Blood specimens were stored at −80 °C after blood collection. Information on lifestyle variables, including smoking habits, was extracted from the questionnaires.

NSHDS is an ongoing prospective cohort and intervention study in Västerbotten County in northern Sweden. Study participants have been invited to participate since 1985 by attending a health check‐up at 40, 50 and 60 years of age [26]. At the health check‐up, participants were asked to complete a self‐administered questionnaire including various demographic factors such as education, smoking habits, physical activity, and diet. In addition, height and weight measurements and blood specimens were collected. Participants diagnosed with LC cases until 4 years after donation of blood specimens were identified through linkage to the regional cancer registry. One control was chosen at random for each case matched on date of birth, ethnicity, gender, date of blood collection, and smoking status.

### 2.2 Biological specimens, RNA isolation, RNA processing, and sequencing

We used diagnostic serum specimens from NLCB, pre‐diagnostic serum specimens from HUNT, and pre‐diagnostic plasma specimens from NOWAC and NSHDS. All specimens were subjected to small RNA extraction. The processing of specimens included the preparation of libraries to target miRNAs specifically before specimens were analyzed using sequencing chips produced by Illumina. Laboratory processing included isolation and purification of miRNA from either serum or plasma and was performed at Biobank1, St. Olavs Hospital, Trondheim, Norway. The starting material was 100 μL serum (NLCB, HUNT) or 100 μL plasma (NOWAC, NSHDS). The miRNeasy Serum/Plasma Kit (Cat No./ID: 217184, Qiagen, Hilden, Germany) in combination with an automated Qiacube system, was used to purify total RNA, including miRNA, from plasma and serum. Aliquots of an in‐house pooled reference specimen (plasma from 10 random healthy individuals) were included in all specimen preparation batches to allow for the evaluation of variation across batches.

Preparation of small RNA sequencing libraries and sequencing experiments were performed at the Genomics Core Facility at NTNU, Trondheim, Norway, in separate batches per cohort study. For assessment of extracted RNA quality and relative size, selected specimens were measured using the Agilent Eukaryote Total RNA Pico assay and an Agilent 2100 Bioanalyzer (Waldbronn, Germany). The sequencing libraries were prepared using extracted RNA eluted in 14 μL water and the NEXTflex small RNA‐seq kit v3 (Bioo Scientific, Austin, TX, USA) according to the manufacturer's instructions. Following adapter ligation and reverse transcription, double‐stranded cDNA was prepared by PCR amplification (22 cycles). Fragments/libraries were run on Agilent 2100 Bioanalyzer (Agilent Technologies, Waldbronn, Germany) or LabChip GX DNA High Sensitivity (Perkin Elmer, Waltham, MA, USA) for quality control and quantitation.

Individual libraries were normalized to 10 nm, pooled, and purified with the QIAquick PCR Purification Kit (Qiagen AB, Stockholm, Sweden) according to instructions. Automated size selection was performed using the Blue Pippin (Sage Science, Beverly, MA, USA), with a range of 135 to 175 bp to select ~152 bp miRNA fragments. Following size selection, the pool was evaluated on Bioanalyzer (Agilent Technologies, Santa Clara, CA, USA) using the High Sensitivity DNA kit. The pool of libraries was quantified with the KAPA Library Quantification Kit (Roche, Pleasanton, CA, USA). Quantitated libraries were further diluted and normalized to 2.4 nm before clustering on the cBot (Illumina, Inc., San Diego, CA, USA). Single read sequencing was performed for 51 cycles on four HiSeq4000 flowcells (one per cohort study), according to the manufacturer's instructions (Illumina, Inc.). Sequence reads were demultiplexed and converted from BCL to fastq file format using bcl2fastq2 conversion software V2.20.0422 (Illumina, Inc.).

### 2.3 Processing of sequence data

The sequence data were processed as outlined by Farazi et al. [27] and included the following steps: adapters from the 3' end of the raw sequences were trimmed using cutadapt‐1.2.1 [28]. The trimmed sequence were collapsed into single unique reads along with their total read count using the fastx collapser tool (http://hannonlab.cshl.edu/fastx_toolkit/) and mapped to the human (hg38) genome using bowtie2 [29], allowing for up to 10 alignments per read to account for reads from duplicated miRNA loci. Reads overlapping with mature miRNA loci were identified using htseq‐count from the htseq python package [30]. These reads were further filtered to identify those with perfect alignment to the genome, and the total read counts for mature miRNAs were then computed by summing the total read count per sequence (isomiR) overlapping each miRNA locus. Mature miRNAs and non‐coding RNAs were annotated using miRBase (Release 21, 2014) [31] and RNA Central (http://rnacentral.org), respectively.

The data were stored and analyzed using the NTNU HUNT Cloud facilities and r (R Core Team, Vienna, Austria).

### 2.4 Identification of candidate microRNAs in diagnostic specimens

Differentially expressed miRNAs and isomiRNAs were identified using the bioconductor package limma combined with voom transformation [32, 33]. To compare miRNA expression between specimens, read counts were normalized using the calibrator RNA normalization factors calculated in limma, followed by reads per million (RPM) normalization. The calibrator RNAs were not filtered prior to normalization and the calcNormFactors in limma were calculated using the full calibrator count matrix. MiRNAs with average RPM < 1 were excluded from the statistical analyses. Correlations of detectable mature miRNAs in the in‐house pooled reference specimens were assessed to ensure comparability of results from the different specimen preparation batches.

We defined candidate miRNAs as differentially‐expressed, mature miRNAs that had (a) log fold changes (log_2_ FC) > 1 between all LC cases and FalsePos, SCLC and FalsePos, or late‐stage LC and FalsePos; i.e. upregulated in LC cases; (b) Bonferroni adjusted *P*‐values < 0.05; and (c) average expression > 5 log_2_ RPM as a technical threshold. We focused on miRNAs that were upregulated in LC cases compared with FalsePos, as we deemed miRNAs with elevated signals as more robust against technical artifacts in a clinical setting than those with downregulated signals. All models used to identify candidate miRNAs included case status (LC case or FalsePos), stage status (early‐, middle‐, or late‐stage LC), histological subtype (SCLC, AD, SQ, Other, not available), age (scaled as (observations − mean)/standard deviation), sex, and lane on sequencing chip, and presented *P*‐values were corrected for false‐discovery rates (FDR). We included age (scaled as previously mentioned), sex, and lane on sequencing chip as covariates in linear models (r package *limma*) using variance‐stabilized counts (using *voom* transformation) normalized to RPM. We also estimated odds ratios (ORs) for LC for the group differences between all LC cases and FalsePos, SCLC and FalsePos, NSCLC and FalsePos, and late‐stage LC and FalsePos using logistic regressions adjusted for age (scaled), sex, and lane on sequencing chip. Survival models included vital status (follow‐up for LC death until August 2018), stage status (early‐, middle‐, late‐stage LC), histological subtype (NSCLC, SCLC), age (scaled as previously mentioned), sex, and smoking status (never, former, current) in the *Surv* function in the r package *survival*. The number of individuals included in each model varied due to varying number of missing information in included covariates in the respective models. Adjusted *P*‐values were obtained using the *p.adjust* function specifying Bonferroni adjustment.

### 2.5 Evaluation of candidate microRNAs in pre‐diagnostic specimens

We evaluated the presence of candidate miRNAs in pre‐diagnostic specimens by focusing on differential expression between LC cases and matched controls. We used mixed effects logistic regression models (*glmer* function in the r package *lme4*), adjusted for age (scaled as previously mentioned) and sex, in addition to a random effect for the three studies included in our prospective study sample to account for study‐specific effects, and ORs were estimated. We also carried out sensitivity analyses that included additional adjustment for smoking status (never, former, and current) or pack‐years, and that restricted the models to cases diagnosed within 2 years of specimen collection. The number of individuals included in each model varied due to varying number of missing information in included covariates in the respective models.

For further evaluation of temporal variation of miRNA signals, generalized additive models were employed (*gam* function in the r package *gam*) and included spline regression for time in days between specimen collection and LC diagnosis with three degrees of freedom in addition to age (scaled as previously mentioned), sex, and smoking status (never, former, and current). In these models, the miRNA signal was represented by residuals from a mixed model including RPM values for each miRNA in a model including the main matching factors age (scaled as previously mentioned), sex, and smoking status (never, former, and current), in addition to a random effect for the three prospective studies to account for study‐specific effects.

### 2.6 Evaluation of diagnostic and predictive values of candidate microRNAs

The ability of candidate miRNAs to distinguish between LC cases and FalsePos in the hospital‐based study sample, and between LC cases and matched controls in the prospective study sample, was investigated using receiver operating characteristic (ROC) curves (*roc* function in the r package *pROC*). This ability was then compared with that of smoking information alone, using models that were adjusted for smoking status in the hospital‐based study sample and for pack‐years in the prospective study sample.

### 2.7 Assessment of candidate microRNA expression in lung tissue using the TCGA database

To assess expression of the candidate miRNAs in lung tissue we used sequence‐based miRNA data available from The Cancer Genome Atlas (TCGA) project using the bioconductor package TCGABiolinks [34, 35]. We analyzed differences in miRNA expression in normal lung tissue compared with lung tumor tissue (AD or SQ, i.e., TCGA‐LUAD or TCGA‐LUSC, respectively). We also analyzed whether miRNA expression in tumor tissue differed across pathological stages I–IV (information available in TCGA). Logistic regression models tested differences in expression between tumor tissue and normal tissue for candidate miRNAs using log2 transformed count values. Linear regression models (glm function in base r) tested differences in expression related to cancer stage in tumor tissue and those models included sex and age at baseline (scaled as previously mentioned) as covariates.

## 3 Results

### 3.1 Study sample

The final hospital‐based study sample included 128 LC cases and 62 FalsePos; the final prospective study sample included 266 LC cases and matched controls from NOWAC, 258 from the NSHDS, and 238 from HUNT (Table 1).

**Table 1:** Main characteristics of participants in the hospital‐based and prospective study samples. AD, adenocarcinoma LC; ES, early‐stage LC; FalsePos, false positives; HUNT, the Trøndelag Health Study; LS, late‐stage LC; MS, middle‐stage LC; NA, not available; NLCB, the Norwegian Lung Cancer Biobank; NOWAC, the Norwegian Women and Cancer Study; NSHDS, the Northern Sweden Health and Disease Study; Other LC, other histological subtypes of LC; SCLC, small cell LC; SQ, squamous cell carcinoma LC.

	Hospital‐based study sample, Diagnostic specimens				Prospective study sample, Pre‐diagnostic specimens					
	NLCB, *n* = 190				NOWAC, *n* = 266		NSHDS, *n* = 258		HUNT, *n* = 238	
	FalsePos	ES	MS	LS	Cases	Controls	Cases	Controls	Cases	Controls
*n*	62	23	42	63	133	133	129	129	119	119
Sex										
Women	26	9	23	22	133	133	67	67	42	42
Men	36	14	19	41	0^a^	0^a^	62	62	77	77
Smoking status										
Never	11	1	0	5	14	57	14	46	0^a^	0^a^
Former	30	11	29	35	37	35	42	47	37	33
Current	21	11	13	23	82	41	67	31	80	85
NA	‐	‐	‐	‐	‐	‐	6	5	2	1
Histological subtype										
SCLC	‐	0	4	16	38	‐	13	‐	40	‐
AD	‐	5	10	27	69	‐	51	‐	39	‐
SQ	‐	10	14	11	19	‐	30	‐	40	‐
Other LC	‐	8	14	9	7	‐	13	‐	‐	‐
NA	‐	0	0	0	0	‐	22	‐	‐	‐
Stage status										
ES	‐	23	‐	‐	30	‐	25	‐	16	‐
MS	‐	‐	42	‐	33	‐	28	‐	32	‐
LS	‐	‐	‐	63	70	‐	56	‐	58	‐
NA	‐	‐	‐	‐	0	‐	20	‐	13	‐
Year of diagnosis										
Mean	‐		2009		2009	‐	2005	‐	2006	‐
Min	‐		2006		2004	‐	1989	‐	1998	‐
Max	‐		2012		2011	‐	2017	‐	2005	‐
Years between specimen collection and LC diagnosis										
Mean	‐	‐	‐	‐	3.81	‐	2.91	‐	4.57	‐
Min	‐	‐	‐	‐	0.003	‐	0.11	‐	0.08	‐
Max	‐	‐	‐	‐	7.92	‐	5	‐	8.22	‐
<2	‐	‐	‐	‐	33	‐	29	‐	25	‐
NA	‐	‐	‐	‐	‐	‐	13	‐	‐	‐
Age at specimen collection										
Mean	63.3	68.1	70.8	66.8	57.0	57.0	57.5	57.4	63.3	63.2
Min	31.8	51.3	49.3	45.5	48.0	48.0	40.0	40.0	34.6	34.6
Max	85.5	82.0	85.2	86.7	63.0	63.0	70.8	70.6	87.6	86.2

^a^ This group was not included as eligible participants in this study.

### 3.2 Identification and characteristics of candidate microRNAs in diagnostic specimens

**Fig. 1:**
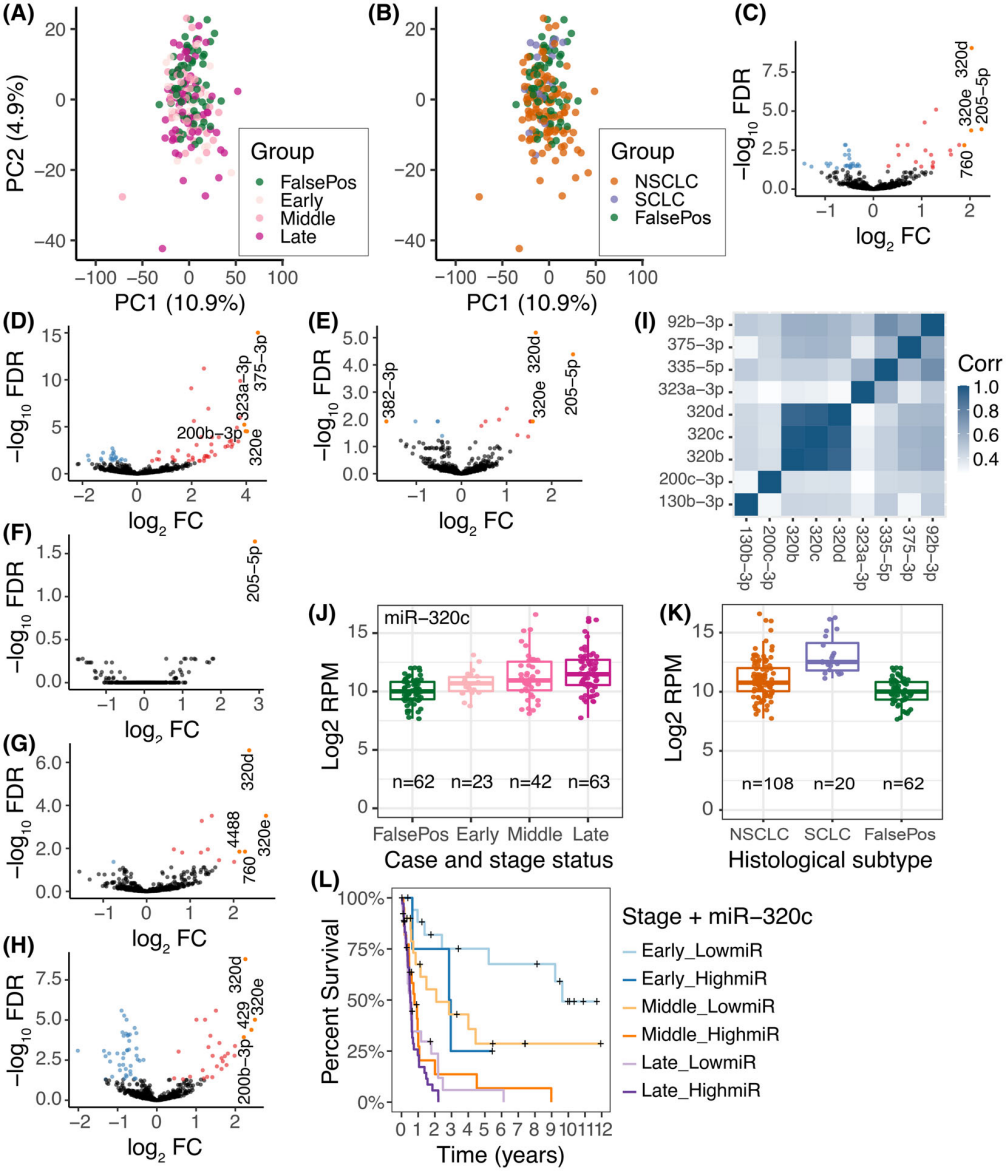
Identification and characteristics of candidate microRNAs (miRNAs) in diagnostic specimens. (A, B) principal component analyses (PCA) plots of miRNA expression for controls (individuals with suspected LC but negative LC diagnostic evaluation; FalsePos) and (A) lung cancer (LC) stage status and (B) histological subtype. (C–H) volcano plots of differentially expressed miRNAs for (C) all LC cases, (D) small cell lung cancer, and (E) non‐small cell lung cancer (NSCLC); and (F) early‐, (G) middle‐, and (H) late‐stage LC compared with FalsePos. All models were adjusted for age, sex, and lane on sequencing chip; estimates are represented as log_2_ fold changes (FC; *x*‐axis) with −log_10_ false discovery rate (FDR)‐adjusted *P*‐values (*y*‐axis). (I) Heatmap of Pearson's correlation coefficients between log_2_ read per million (RPM) values for the candidate miRNAs at diagnosis. (J, K) distribution of log_2_ RPM values for miR‐320c grouped by (J) case status and stage status, and (K) histological subtype. (L) Survival curves for early‐, middle‐ and late‐stage LC subdivided by low or high expression of miR‐320c (indicated as ‘LowmiR’ or ‘HighmiR’, defined as having RPM expression below or above the median RPM value of miR‐320c, respectively); miR‐320c *P* = 0.03 (cox model with stage status, histological subtype, age, sex, and smoking status as covariates; Bonferroni adjusted). Samples included in comparisons are presented in plot or in Table 1 and Tables S3 and S5–S9.

Following sequencing and quality control (Figs S1–S4), our preprocessed and annotated count matrix contained a total of 1663 miRNAs, of which 725 had an average expression > 1 RPM. Principal component analysis of these 725 miRNAs demonstrated no clear separation according to case status, stage status, or histological subtype (Fig. 1A,B), but linear models did show that multiple miRNAs were differentially expressed between LC cases and FalsePos (Fig. 1C), as well as between SCLC/NSCLC and FalsePos (Fig. 1D,E; Table S2). The number of differentially expressed miRNAs increased with more advanced stage status (Fig. 1F–H and Fig. S5), and the largest differences in log_2_ FC were observed when comparing SCLC to FalsePos (16% of all LC cases were SCLC, and 49% of all LC cases and 80% of SCLC were late‐stage; Table 1). Further analyses of the histological subtypes of NSCLC (AD, SQ, and other) did not demonstrate significant results (results not shown).

According to our criteria (log_2_ FC > 1; Bonferroni adjusted *P* < 0.05; log_2_ RPM > 5), nine of the 725 investigated miRNAs were considered candidate miRNAs: miR‐320d, miR‐320c, miR‐320b, miR‐92b‐3p, miR‐130b‐3p, miR‐200c‐3p, miR‐375‐3p, miR‐335‐5p, and miR‐323a‐3p (estimates in all LC cases, late‐stage LC, and SCLC are presented in Table S3). Note that these criteria excluded the early‐stage‐associated miR‐205‐5p (Fig. 1F), because of its low expression (average log_2_ RPM 3.5). Effect estimates in the linear models were similar when additionally adjusted for smoking status (Table S4). No miRNA was significantly associated with smoking status.

Except for the three miRNAs from the miR‐320 family, the candidate miRNAs demonstrated limited expression correlation (Fig. 1I). When comparing LC cases and FalsePos, miR‐320b, miR‐320c, and miR‐320d demonstrated the largest differences in average expression and showed the highest ORs (2.46, 95% CI 1.83–3.32; 2.51, 95% CI 1.77–3.54; and 2.85, 95% CI 1.91–4.26, respectively; Table S5). These ORs were larger for late‐stage LC or SCLC (Tables S6–S8). For NSCLC, the increased OR was driven by a subset of middle‐ and late‐stage LC expressing higher levels of miR‐320 miRNAs than any FalsePos (Fig. 1J,K). Higher expression of all three miR‐320 miRNAs was also associated with poorer survival, with middle‐stage LC having the largest separation in survival curves for low and high expression of miR‐320c (Fig. 1L and Fig. S6). For miR‐320c, high expression compared with low was significant when adjusting for age, sex, smoking status, LC stage and LC histology (Table S9). These results demonstrated that circulating miR‐320 miRNAs are potential markers of the presence of advanced (late‐stage or SCLC) LC.

### 3.3 Evaluation of candidate microRNAs in pre‐diagnostic specimens

As in the diagnostic specimens, miR‐320b, miR‐320c, and miR‐320d were the most strongly correlated candidate miRNAs in pre‐diagnostic specimens, though the correlation patterns varied across the three studies included in our prospective study sample (Fig. S7). None of the candidate miRNAs were differentially expressed between LC cases and matched controls when considering all LC, late‐stage LC, SCLC, or NSCLC, irrespective of time between specimen collection and diagnosis (Fig. S8; Tables S5–S8); adjusting for smoking exposure had negligible effects on the models.

**Fig. 2:**
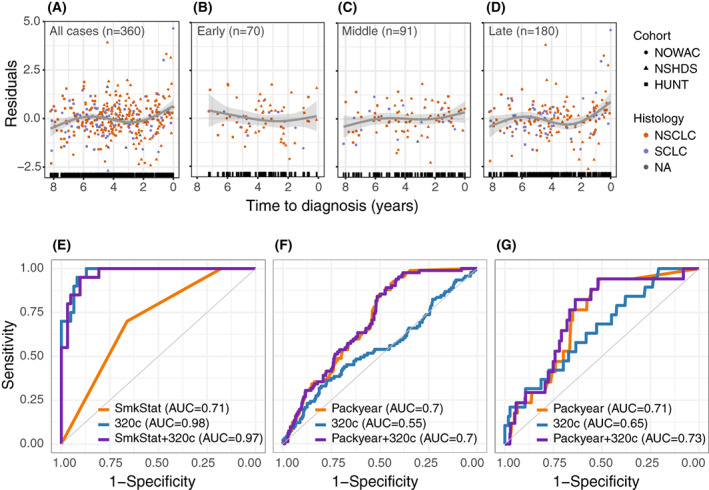
Expression and predictive power of miR‐320c in pre‐diagnostic specimens. (A–D) representation of trends for expression levels for miR‐320c pre‐diagnostic specimens from LC cases in the prospective study sample (including NOWAC, NSHDS, and HUNT) according to the number of years between specimen collection and diagnosis for (A) all lung cancer (LC) cases, (B) early‐stage LC, (C) middle‐stage LC, and (D) late‐stage LC. Specimens are colored according to histological subtype (SCLC, Small cell lung cancer; NSCLC, Non‐small cell lung cancer). The observations plotted represent residuals from mixed models including log_2_ read per million (RPM) values that were adjusted for matching factors (age, sex, smoking status) in addition to a random effect for the three studies included in our prospective study sample, to account for study‐specific effects. Predicted trends for splines with three degrees of freedom for years between specimen collection and diagnosis in a generalized additive model are indicated in dark gray; confidence intervals are in light gray. (E–G) receiver operating characteristic (ROC) curves for (E) LC case‐FalsePos discrimination for specimens from SCLC in the hospital‐based study sample (NLCB, *n* = 20 SCLC, 62 FalsePos), (F) LC case–control discrimination in the prospective study sample (NOWAC, NSHDS, HUNT, *n* = 91 SCLC, 375 controls), and (G) LC case–control discrimination in the prospective study sample in pre‐diagnostic specimens collected within 2 years of LC diagnosis (*n* = 19 SCLC, 375 controls). Separate ROC curves are displayed for models including smoking status (SmkStat) for the hospital‐based study sample or pack‐years (packyear) for the prospective study sample, miR‐320c expression only (320c), and both the smoking variable and miR‐320c expression (SmkStat + 320c, packyear + 320c). HUNT, Trøndelag health study; NLCB, Norwegian lung cancer biobank; NOWAC, Norwegian women and cancer study; NSHDS, Northern Sweden health and disease study.

When modeling candidate miRNA expression in relation to time between specimen collection and diagnosis, we found that miR‐320c and miR‐320d had higher expression in blood specimens collected closer to diagnosis (Fig. 2A and Fig. S9; Table S10). Specifically, the model fit indicated increasing expression of miR‐320c and miR‐320d within 2 years of diagnosis, though this trend appeared to be primarily driven by specimens from cases with late‐stage LC and SCLC (Fig. 2B–D and Fig. S9; Tables S11–S13).

Restricting case–control comparisons to cases with specimens collected within 2 years of LC diagnosis increased the estimated ORs for all the miR‐320 candidates, with miR‐320d reaching significance for all LC cases (OR 1.25, 95% CI 1.01–1.54; Table S5). Although the OR estimates for miR‐320d were higher for late‐stage LC and SCLC, none of the candidate miRNAs reached statistical significance, likely because of the limited number of cases in those subgroups (Tables S6 and S7). In comparison, the ORs for miR‐320c were similar to those for miR‐320d in all LC cases, late‐stage LC, and SCLC diagnosed within 2 years of specimen collection, and reached statistical significance for NSCLC diagnosed within 2 years of specimen collection (OR 1.41, 95% CI 1.00–1.99; Table S8). These results suggest that certain candidate miRNAs were indicative of LC close to time of diagnosis.

### 3.4 Evaluation of the diagnostic and predictive value of candidate microRNAs for lung cancer

To evaluate the ability of candidate miRNAs to discriminate between LC cases and FalsePos/controls, we compared the models that included candidate miRNAs to those based on smoking information alone. In the hospital‐based study sample, miR‐320c or miR‐320d expression was a much better predictor of LC than smoking status alone (area under the curve, AUC 0.74, 0.80, and 0.55, respectively). Discrimination of miR‐320c was slightly higher for late‐stage LC (AUC 0.78) and markedly higher for SCLC (AUC 0.98; Fig. 2E). In the prospective study sample, however, smoking exposure (pack‐years) was a better predictor of LC development than miR‐320c or miR‐320d (AUC 0.61, 0.53, and 0.53, respectively; Fig. S10). For SCLC, discriminative power of smoking exposure was higher but similar for miR‐320c (AUC 0.70 and 0.55, respectively; Fig. 2F). Restricting to cases diagnosed within 2 years of specimen collection had little effect on the discriminative power of smoking exposure (AUC 0.62), but slightly improved that of miR‐320c (AUC 0.58). Restricting the analyses to late‐stage LC or NSCLC gave similar results, but for SCLC, the AUC for miR‐320c improved from 0.55 in SCLC cases to 0.65 in SCLC diagnosed within 2 years of specimen collection (Fig. 2G). Overall, these results suggest that the candidate miRNAs had limited potential to predict LC development long before diagnosis, but that miR‐320c and miR‐320d were indicative of LC close to and at diagnosis.

### 3.5 Expression of candidate microRNAs in lung tissue using the TCGA database

We analyzed miRNA expression in 519 tumor and 46 normal samples, and 478 tumor and 45 normal samples for AD and SQ subtype datasets, respectively. All candidate miRNAs were available in the TCGA database. In both datasets, miR‐375 was the most highly expressed of the miRNA candidates and the miR‐320s were lowly expressed in both datasets (Tables S14 and S15). When compared with normal tissue, there was higher expression of all candidate miRNAs in AD tumor tissue and of two miRNAs in SQ tumor tissue. Further, there was lower expression of five candidate miRNAs in the SQ tumor tissue. The largest expression differences were observed for miR‐130b in both AD and SQ lung tissue (AD presented in Fig. S11A). There were no significant miRNA expression differences across stages for AD (minimum *P*‐value = 0.13), but for SQ *P*‐values for differences between stages were 0.02 and 0.04 for miR‐130b and miR‐200c, respectively. Still, no trend was apparent (miR‐130b is presented in Fig. S11B).

## 4 Discussion

The novelty of our study lies in investigating how long before LC diagnosis case–control differences in candidate miRNAs could be detected. We approached this by first identifying candidate miRNAs in diagnostic specimens from a hospital‐based study sample, and then evaluating expression of these candidate miRNAs in pre‐diagnostic specimens taken up to 8 years prior to LC diagnosis from a prospective study sample from three, population‐based studies. Among more than 1600 miRNAs analyzed, nine candidate miRNAs were identified in the diagnostic specimens. LC associations were strongest and most consistent in both the hospital‐based and the prospective study samples for two candidate miRNAs: miR‐320c and miR‐320d. In the hospital‐based study sample, increased expression of miR‐320c and miR‐320d was associated with poor survival, and high discriminative ability was observed for SCLC. Further, the expression of miR‐320c and miR‐320d was upregulated in LC cases with pre‐diagnostic specimens collected within 2 years of diagnosis when compared with the matched controls, especially for late‐stage LC and SCLC. Therefore, monitoring these miRNAs could have a clinical impact by indicating individuals at high risk or who should have shorter screening intervals.

Expression of the miR‐320 family in blood has been previously associated with LC [14, 36, 37, 38]. Indeed, miR‐320 is included in the blood‐based miRNA panel, ‘circulating miRNA signature classifier’ (MSC), which has recently demonstrated promising predictive value for LC incidence in a screening setting [13]. Although the associations with the miR‐320 family were strongest for SCLC in our study, they were also observed for late‐stage LC and NSCLC. As the majority of SCLC occurred in patients with late‐stage disease, we cannot disregard that these miRNAs might primarily relate to advanced disease *per se*. As such, they might not be specific to LC. Upregulated miR‐320 expression has been observed in metastatic or late‐stage colorectal cancer [39, 40, 41], and members of the miR‐320 family were the strongest individual predictors for 12 [42] or 13 [43] different cancer types, including LC. Further, miR‐320 signals in blood could be informative of cancer risk before the manifestation of advanced LC in patients with chronic obstructive pulmonary disease [44] and may also have relevance for predicting treatment strategies, as patients with advanced NSCLC and elevated blood expression of miR320b‐d showed poorer outcomes following immunotherapy [45].

The expression of candidate miR‐320s was relatively low in both AD and SQ lung tumor tissue and there were no clear differences between tumor and normal lung tissue samples. Elevated miR‐320 expression in plasma extracellular vesicles has been reported to have pro‐tumorigenic activity [46]. Still, it is not known how blood expression patterns correlate with those in lung tumor tissue, where downregulation of anti‐tumorigenic activity by miR‐320 family members has been observed [47, 48, 49]. The miR‐320 family has been shown to have a function in the regulation of genes involved in cell growth, migration, and invasion [37]. Moreover, miR‐320 expression can reflect systemic inflammation or altered immune responses, as miR‐320 has been associated with immunosuppressive and protumorigenic blood and tumor phenotypes and future LC risk [21, 50]. Whether the observed and reported associations with LC reflect the miR‐320 family's general role in cancer‐related molecular functions, systemic inflammation, or altered immune responses, are open questions.

Other than the miR‐320 family, there is limited overlap between our candidate miRNAs and miRNAs that have been previously associated with LC. Notable exceptions include: miR‐200c‐3p, which was linked to NSCLC [17] and to early‐stage NSCLC [18]; miR‐375, which was linked to both SCLC and NSCLC [51]; and miR‐130c‐3p (closely related to miR‐130b‐3p), which was linked to NSCLC [19]. Although miRNAs are promising blood‐based markers of LC [12, 14, 16, 17, 18, 19, 36], the overlap of reported miRNAs between studies is limited. Heterogeneity in analytical technology, the number of miRNAs quantified, and the statistical methods used likely contribute to the discrepancies in results across studies. Still, expression of miR‐130b was higher in lung tumor tissue compared with normal lung tissue in the TCGA data. Higher blood miR‐130b expression observed in LC cases in the NLCB study might be in agreement with higher miR‐130b expression in lung tumor tissue in the TCGA dataset. However, elevated miR‐130b expression was not indicated in pre‐diagnostic blood samples.

The majority of studies exploring the diagnostic, prognostic, or predictive value of miRNAs have included specimens taken at diagnosis. There are few studies based primarily on pre‐diagnostic specimens, and although these have reported miRNAs to be differentially expressed at different time periods prior to LC diagnosis [20, 21, 22, 23], it is unclear to what extent these reported statistical signals reflect LC disease development or represent altered LC risk. Notably, two blood‐based miRNA panels, miR‐Test [12] and MSC [14, 21], were developed based on specimens collected prior to and at diagnosis. Recent results showed that using the MSC panel in combination with low‐dose computed tomography scans had increased ability to predict individual LC incidence and mortality compared with these scans alone in the large Multicentric Italian Lung Detection screening trial, which included over 4000 heavy smokers [13]. Our results showed that miR‐320c and miR‐320d could be indicative of imminent and advanced LC in pre‐diagnostic specimens as well.

Our study design and novel approach allowed us to identify miRNAs that were differentially expressed at the time of LC diagnosis, as well as up to 8 years prior to LC diagnosis. However, our prospective study sample included participants from three distinct studies; thus, we evaluated differences in miRNA expression among individuals with different times between specimen collection and LC diagnosis. Future studies should include repeated measurements prior to LC diagnosis, as they could contribute to the understanding of whether the observed signals are related to pre‐disease conditions or to present, but clinically undetected LC. Based on our results, such studies should include annual specimen collections to capture relevant miRNA expression changes both at and prior to LC diagnosis.

Although tobacco smoking is a strong predictor of future LC risk, many never smokers also get LC, so relevant markers for LC should ideally be unrelated to tobacco exposure. In this study, we observed little influence of smoking status on model estimates for the miR‐320 family members, indicating that these markers likely did not reflect past exposure to tobacco smoking. Previous studies are both in agreement [52] and disagreement [53] with our observations.

Our study has several strengths compared with previous studies. First, the combination of diagnostic and pre‐diagnostic specimens allowed us to evaluate potential miRNAs both at and prior to LC diagnosis. Second, we were able to compare the expression profiles of LC cases to those of individuals with suspected LC but negative LC diagnostic evaluation (FalsePos), rather than to healthy controls. We believe that such ‘symptomatic controls’ are better suited than healthy controls to identify markers for use in clinical settings or for screening high‐risk groups. Third, we did not exclude any LC stages or histological subtypes, thus our study represents a realistic distribution in a screening setting, in contrast to many studies that have focused specifically on NSCLC and early‐stage LC. Fourth, we chose to explore candidate miRNAs that were upregulated in LC cases, as elevated signals represent a more realistic measure in relation to LC prediction. Finally, we observed the reported associations in both plasma and serum specimens, which is of importance as it generalizes the results across sample materials.

One major limitation of this study is that the selection criteria for LC cases, as well as the matching criteria for controls, differed across the three studies included in our prospective study sample. Another potential limitation is that the smoking information retrieved from the medical records of LC patients in the hospital‐based study sample could be biased when compared with the information in the prospective study sample, as NLCB participants reported their smoking status during clinical follow‐up. Further, the higher proportion of SCLC and late‐stage LC in the hospital‐based compared with the prospective study sample (SCLC was enriched by design in HUNT) may have contributed to candidate miRNAs being less prominent in the prospective study sample. Still, the different designs and participants included in the four studies that comprised our study samples suggest that the signals we did observe were robust and will generalize beyond our study samples. Of note, blood specimens in the hospital‐based study sample were obtained before immunotherapy was implemented as a standard therapy for advanced NSCLC, so the survival analyses of LC patients reflect a time period prior to these developments and would be different if the specimens had been obtained in recent years.

## 5 Conclusions

We identified nine candidate miRNAs with increased expression in the diagnostic specimens of LC cases compared with false‐positive controls (i.e. those with suspected LC but negative LC diagnostic evaluation). Of these, high expression of miR‐320b, miR‐320c, and miR‐320d was associated with poor survival, independent of LC stage and histological subtype. Moreover, expression of miR‐320c and miR‐320d was elevated in pre‐diagnostic specimens taken from late‐stage LC and SCLC within 2 years of diagnosis. These results indicate that miR‐320c and miR‐320d can be used as early markers of imminent and advanced LC, but that screening intervals should then be less than 2 years.

## Conflict of interest

The authors declare no conflict of interest.

## Author contributions

THN, PS, and TMS conceptualized and designed the study. THN, PS, TMS, AHS, IU, E‐EP, TD, SA, RM, Mikael J, and MM were responsible or contributed to acquisition, analysis, or interpretation of data. THN and PS performed the statistical analysis. TMS, PS, BHG, Mattias J, and ODR provided administrative, technical, or material support. TMS obtained funding. THN, TMS, PS, AHS, and IU drafted the manuscript and all authors read and approved the final manuscript. The work reported in the article has been performed by the authors, unless clearly specified in the text.

## Supporting information


**Fig. S1.** Quality assessment of sequencing experiments for NLCB specimens (*n* = 215 study specimens and eight in‐house pooled reference specimens).
**Fig. S2.** (A) Principal component analysis (PCA) plot displaying variables and specimens for the preprocessed data in NLCB. Specimens indicate lane on sequencing chip and (B) correlation of reads for those miRNAs that were detected > 1 read per million for aliquots of an in‐house pooled reference specimen prepared and analyzed in batches together with NLCB specimens (*n* = 8 aliquots).
**Fig. S3.** Quality assessment of sequencing experiments for (A) NOWAC specimens (*n* = 267 study specimens and eight aliquots of an in‐house pooled reference specimen), (B) NSHDS specimens (*n* = 258 study specimens and eight aliquots of the in‐house pooled reference specimen), and (C) HUNT specimens (*n* = 238 study specimens and eight aliquots of the in‐house pooled reference specimen).
**Fig. S4.** For the preprocessed data in (A) NOWAC, (B) NSHDS, (C) HUNT, correlation of reads for those miRNAs that were detected > 1 read per million for aliquots of an in‐house pooled reference specimen prepared and analyzed in batches together with specimens from (A) NOWAC (*n* = 8 aliqouts), (B) NSHDS (*n* = 8), (C) HUNT (*n* = 8).
**Fig. S5.** Heatmap displaying the relative expression of miRNAs in NLCB, the direction of differential expression and the absolute expression of the same miRNAs in order of appearance from left to right.
**Fig. S6.** High expression of (A) miR‐320b and (B) miR‐320d was associated with lower survival in NLCB (*P* = 0.02 and *P* = 0.03, respectively).
**Fig. S7.** Heatmap of Pearson's correlation coefficients for log2 read per million values for candidate miRNAs in the three pre‐diagnostic studies (NOWAC, NSHDS, HUNT) combined.
**Fig. S8.** The distribution of log2 reads per million values for one selected candidate miRNA of interest, miR‐320c, in controls and LC stage groups in upper panels, and histological subtypes in the lower panels for specimens from the (A) NOWAC, (B) NSHDS and (C) HUNT studies.
**Fig. S9.** Representation of trends for miR‐320d for LC cases in the pre‐diagnostic specimens (NOWAC, NSHDS, HUNT) according to the number of days in the interval between time of specimen collection and time of diagnosis for (A) all LC, (B) early‐stage LC, (C) middle‐stage LC, and (D) late‐stage LC.
**Fig. S10.** (A) Case‐FalsePos discrimination for specimens in NLCB (*n* = 128 cases, 62 FalsePos), (B) case–control discrimination in the prospective study sample (NOWAC, NSHDS, HUNT, *n* = 373 cases, 375 controls), and (C) case–control discrimination in the pre‐diagnostic specimens collected from cases within 2 years of LC diagnosis (*n* = 84 cases, 375 controls).
**Fig. S11.** (A) Expression of miR‐130b in normal (*N* = 46) and adenocarcinoma tumor tissue (*N* = 519) in the TCGA dataset, (B) Expression of miR‐130b in squamous carcinoma tumor tissue (*N* = 478) displayed for all pathological stages.
**Table S1.** The definition of cancer stage categories based on information either from the Cancer Registry of Norway (NOWAC and HUNT) or medical records (NLCB and NSHDS).
**Table S2.** The total number of differentially expressed miRNAs (considering an FDR *P*‐value threshold), the maximum log FC, and minimum FDR‐adjusted *P*‐value in the different group tests considered.
**Table S3.** Model summaries for the nine candidate miRNAs in NLCB using R limma analyses. Presented are log2 fold change (logFC) values for the difference in expression between FalsePos and all LC cases (*n* = 190), adjusted *P*‐values, crude *P*‐values, and average expression (AveExpr) for each miRNA.
**Table S4.** Model summaries for the nine candidate miRNAs in NLCB using R limma analyses.
**Table S5.** Model summaries for the nine candidate miRNAs in NLCB, and in NOWAC, NSHDS, and HUNT studies.
**Table S6.** Model summaries for the nine candidate miRNAs in *late‐stage cases* in NLCB, and in NOWAC, NSHDS, and HUNT studies.
**Table S7.** Model summaries of models for the nine candidate miRNAs in *SCLC cases* in NLCB and in NOWAC, NSHDS, and HUNT studies.
**Table S8.** Model summaries of models for the nine candidate miRNAs in *NSCLC cases* in NLCB and in NOWAC, NSHDS, and HUNT studies.
**Table S9.** Model summary for survival model for expression of miR‐320c (scaled) in NLCB (LC cases, *n* = 128).
**Table S10.** Model summaries for the nine candidate miRNAs in generalized additive models^a^ allowing for non‐linear trends in miRNA expression levels across time between blood specimen sampling and time of diagnosis in all cases in NOWAC, NSHDS, and HUNT (*n* = 360).
**Table S11.** Model summaries for the nine candidate miRNAs in generalized additive models^a^ allowing for non‐linear trends in miRNA expression levels across time between blood specimen sampling and time of diagnosis in *late‐stage cases* in NOWAC, NSHDS, and HUNT (*n* = 180).
**Table S12.** Model summaries for the nine candidate miRNAs in generalized additive models^a^ allowing for non‐linear trends in miRNA expression levels across time between blood specimen sampling and time of diagnosis in *SCLC cases* in NOWAC, NSHDS, and HUNT (*n* = 91).
**Table S13.** Model summaries for the nine candidate miRNAs in generalized additive models^a^ allowing for non‐linear trends in miRNA expression levels across time between blood specimen sampling and time of diagnosis in *NSCLC cases* in NOWAC, NSHDS, and HUNT (*n* = 269).
**Table S14.** Model summaries for the expression of candidate miRNAs in tumor (*N* = 519) or normal (*N* = 46) lung tissue registered for adenocarcinoma in TCGA databases.
**Table S15.** Model summaries for the expression of candidate miRNAs in tumor (*N* = 478) or normal (*N* = 45) lung tissue registered for squamous cell carcinoma in TCGA databases.

## Data Availability

The normalized count matrix of expressed miRNAs in the NLCB cohort is available in the Gene Expression Omnibus repository (GSE188232; https://www.ncbi.nlm.nih.gov/geo/query/acc.cgi?acc=GSE188232). The raw sequencing data generated and/or analyzed in the prospective cohorts could be accessed upon reasonable request to the originating cohorts. Access will be conditional to adherence to local ethical and security policies. R codes used for the analyses presented in the paper are available upon request.
