# Supplementary material for: Increased levels of microRNA‐320 in blood serum and plasma is associated with imminent and advanced lung cancer
Source: Mol Oncol. 2022 Nov 27;17(2):312–27. doi: 10.1002/1878-0261.13336 (PMC9892825; doi:10.1002/1878-0261.13336)
Supplement: Supplementary file 1 — Fig. S1. Quality assessment of sequencing experiments for NLCB specimens (n = 215 study specimens and eight in‐house pooled reference specimens). Fig. S2. (A) Principal component analysis (PCA) plot displaying variables and specimens for the preprocessed data in NLCB. Specimens indicate lane on sequencing chip and (B) correlation of reads for those miRNAs that were detected > 1 read per million for aliquots of an in‐house pooled reference specimen prepared and analyzed in batches together with NLCB specimens (n = 8 aliquots). Fig. S3. Quality assessment of sequencing experiments for (A) NOWAC specimens (n = 267 study specimens and eight aliquots of an in‐house pooled reference specimen), (B) NSHDS specimens (n = 258 study specimens and eight aliquots of the in‐house pooled reference specimen), and (C) HUNT specimens (n = 238 study specimens and eight aliquots of the in‐house pooled reference specimen). Fig. S4. For the preprocessed data in (A) NOWAC, (B) NSHDS, (C) HUNT, correlation of reads for those miRNAs that were detected > 1 read per million for aliquots of an in‐house pooled reference specimen prepared and analyzed in batches together with specimens from (A) NOWAC (n = 8 aliqouts), (B) NSHDS (n = 8), (C) HUNT (n = 8). Fig. S5. Heatmap displaying the relative expression of miRNAs in NLCB, the direction of differential expression and the absolute expression of the same miRNAs in order of appearance from left to right. Fig. S6. High expression of (A) miR‐320b and (B) miR‐320d was associated with lower survival in NLCB (P = 0.02 and P = 0.03, respectively). Fig. S7. Heatmap of Pearson's correlation coefficients for log2 read per million values for candidate miRNAs in the three pre‐diagnostic studies (NOWAC, NSHDS, HUNT) combined. Fig. S8. The distribution of log2 reads per million values for one selected candidate miRNA of interest, miR‐320c, in controls and LC stage groups in upper panels, and histological subtypes in the lower panels for specimens from the (A) [file MOL2-17-312-s001.docx]

**Supplementary Materials for**

**Increased levels of microRNA-320 in blood serum and plasma is associated with imminent and advanced lung cancer**

Therese Haugdahl Nøst*^1,2^, Anne Heidi Skogholt^2^, Ilona Urbarova^1^, Robin Mjelle^2,3,4^, Erna-Elise Paulsen^5,6^, Tom Dønnem^5,7^, Sigve Andersen^5,7^, Maria Markaki^8^, Oluf Dimitri Røe^3,9^, Mikael Johansson^10^, Mattias Johansson^11^, Bjørn Henning Grønberg^3,12^, Torkjel Manning Sandanger^1^, Pål Sætrom^2,3,4,5,13^

^1^Department of Community Medicine, Faculty of Health Sciences, UiT The Arctic University of Norway, P.O. Box 6050 Langnes, NO-9037 Tromsø, Norway;

^2^K.G. Jebsen Center for Genetic Epidemiology, Department of Public Health and Nursing, NTNU – Norwegian University of Science and Technology, P.O. Box 8905, NO-7491 Trondheim, Norway;

^3^Department of Clinical and Molecular Medicine, NTNU – Norwegian University of Science and Technology, NO-7491 Trondheim, Norway;

^4^Bioinformatics Core Facility, NTNU – Norwegian University of Science and Technology, NO-7491 Trondheim, Norway;

^5^Department of Clinical Medicine, Faculty of Health Sciences, UiT The Arctic University of Norway, NO-9037 Tromsø, Norway;

^6^Department of Pulmonology, University Hospital of North Norway, NO-9038 Tromsø, Norway;

^7^Department of Oncology, University Hospital of North Norway, NO-9038 Tromsø, Norway;

^8^Institute of Computer Science, FORTH, GR-700 13 Heraklion, Crete, Greece;

^9^Cancer Clinic, Levanger Hospital, Nord-Trøndelag Health Trust, NO-7600 Levanger, Norway;

^10^Department of Radiation Sciences, Oncology, Umeå University, SE-901 87 Umeå, Sweden;

^11^International Agency for Research on Cancer, 69372 Lyon CEDEX 08, France;

^12^Department of Oncology, St. Olavs Hospital, Trondheim University Hospital, NO-7030 Trondheim, Norway;

^13^Department of Computer Science, Norwegian University of Science and Technology, NO-7491 Trondheim, Norway.

*Corresponding author: Therese Haugdahl Nøst, UiT The Arctic University of Norway, P.O. Box 6050 Langnes, NO-9037 Tromsø, Norway; E-mail: [therese.h.nost@uit.no](mailto:therese.h.nost@uit.no).


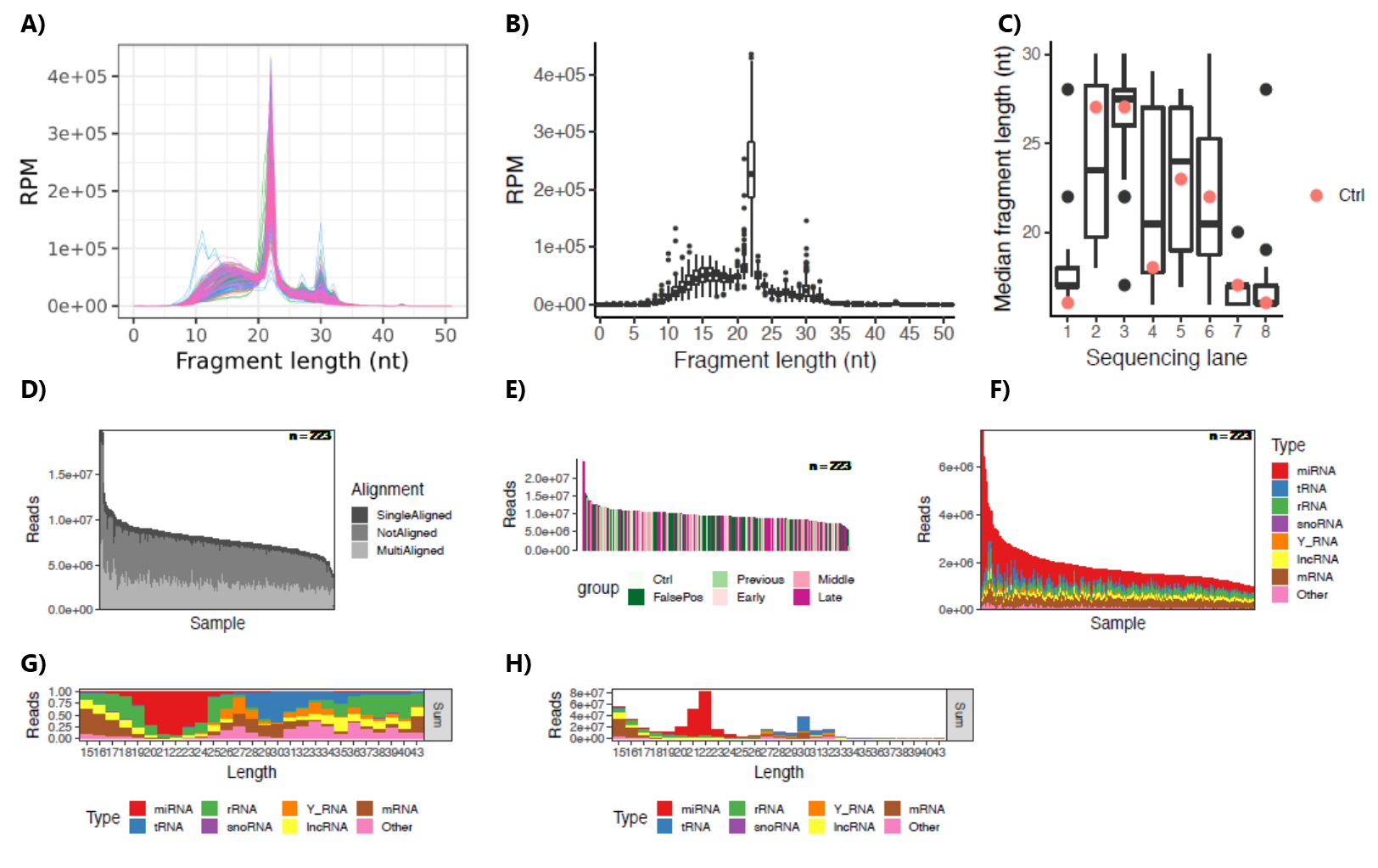


**Figure S1**: Quality assessment of sequencing experiments for NLCB specimens (n=215 study specimens and eight in-house pooled reference specimens. A) Distribution of reads per million (RPM) of fragments according to their fragment length. B) Boxplots of RPM according to fragment length. C) Median fragment lengths according to sequencing lane on the chip in sequencing experiments. D) Reads in specimens sorted according to highest reads on the left and indicated as aligned to a single known sequence (‘SingleAligned’), not aligned to a known sequence (‘NotAligned’) or aligned to multiple known sequences (‘MultiAligned’). E) Reads in specimens colored by specimen group (In-house pooled reference specimens - ‘Ctrl’, Previous cancers, Middle-stage LC, False positives, Early-stage LC, Late-stage LC). F) Reads in specimens colored by type of small RNA (miRNA, tRNA, rRNA, snoRNA, Y_RNA, lncRNA, mRNA, Other). G) Fractions of reads in specimens by type of small RNA (miRNA, tRNA, rRNA, snoRNA, Y_RNA, lncRNA, mRNA, Other). H) Reads in specimens according to fragment length colored by type of small RNA (miRNA, tRNA, rRNA, snoRNA, Y_RNA, lncRNA, mRNA, Other).

**
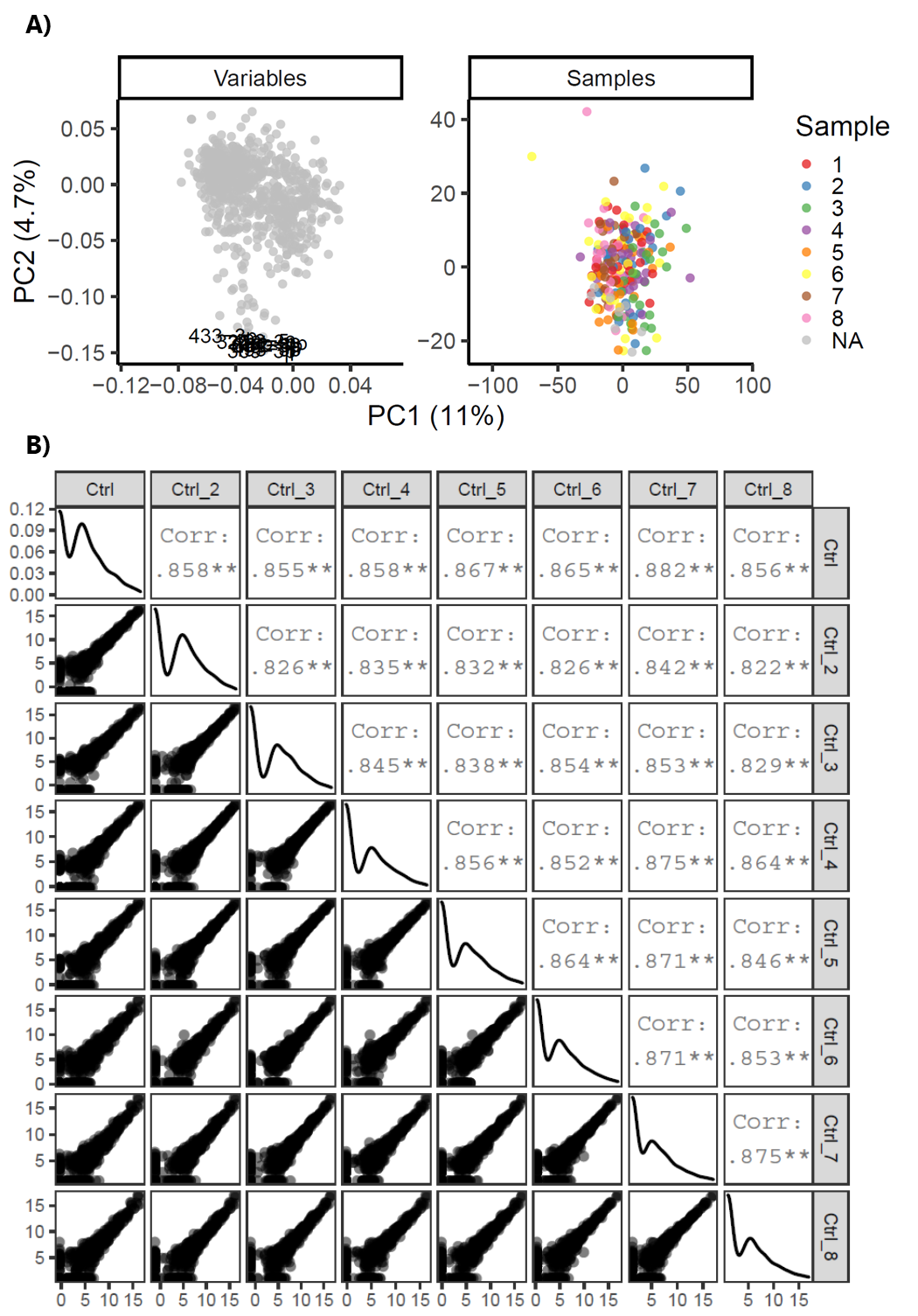
**

**Figure S2**: A) Principal component analysis (PCA) plot displaying variables and specimens for the preprocessed data in NLCB. Specimens indicate lane on sequencing chip and B) correlation of reads for those miRNAs that were detected >1 read per million for aliquots of an in-house pooled reference specimen prepared and analysed in batches together with NLCB specimens (n=8 aliquots).


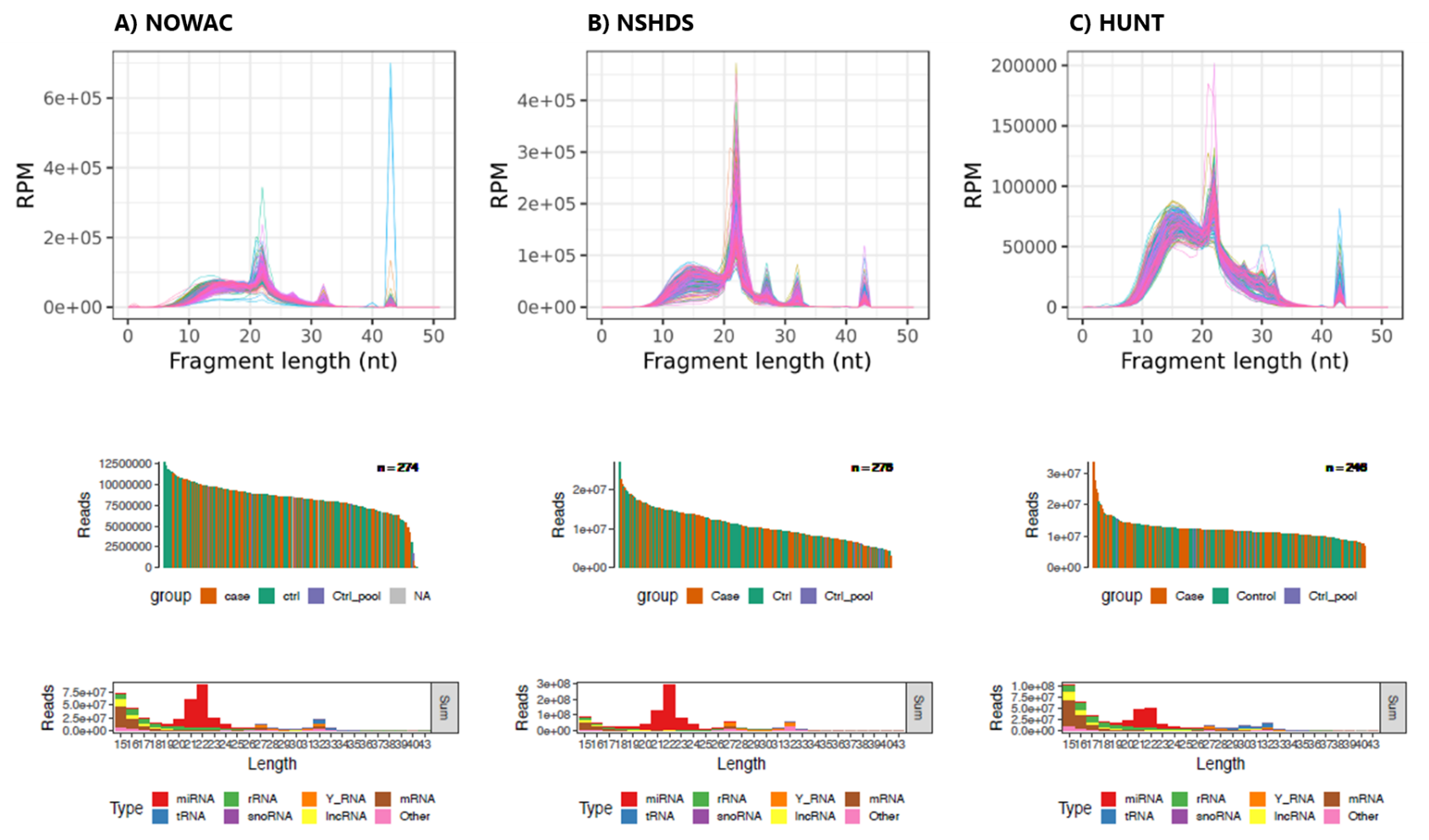


**Figure S3**: Quality assessment of sequencing experiments for A) NOWAC specimens (n=267 study specimens and eight aliquots of an in-house pooled reference specimen), B) NSHDS specimens (n=258 study specimens and eight aliquots of the in-house pooled reference specimen), and C) HUNT specimens (n=238 study specimens and eight aliquots of the in-house pooled reference specimen). The topmost plot displays distribution of read per million of fragments according to their fragment length, the middle plot displays reads in specimens colored by specimen group (case, control or in-house pooled reference specimen ‘Ctrl_pool’), and the bottom plot displays reads in specimens according to fragment length colored by type of small RNA (miRNA, tRNA, rRNA, snoRNA, Y_RNA, lncRNA, mRNA, Other).


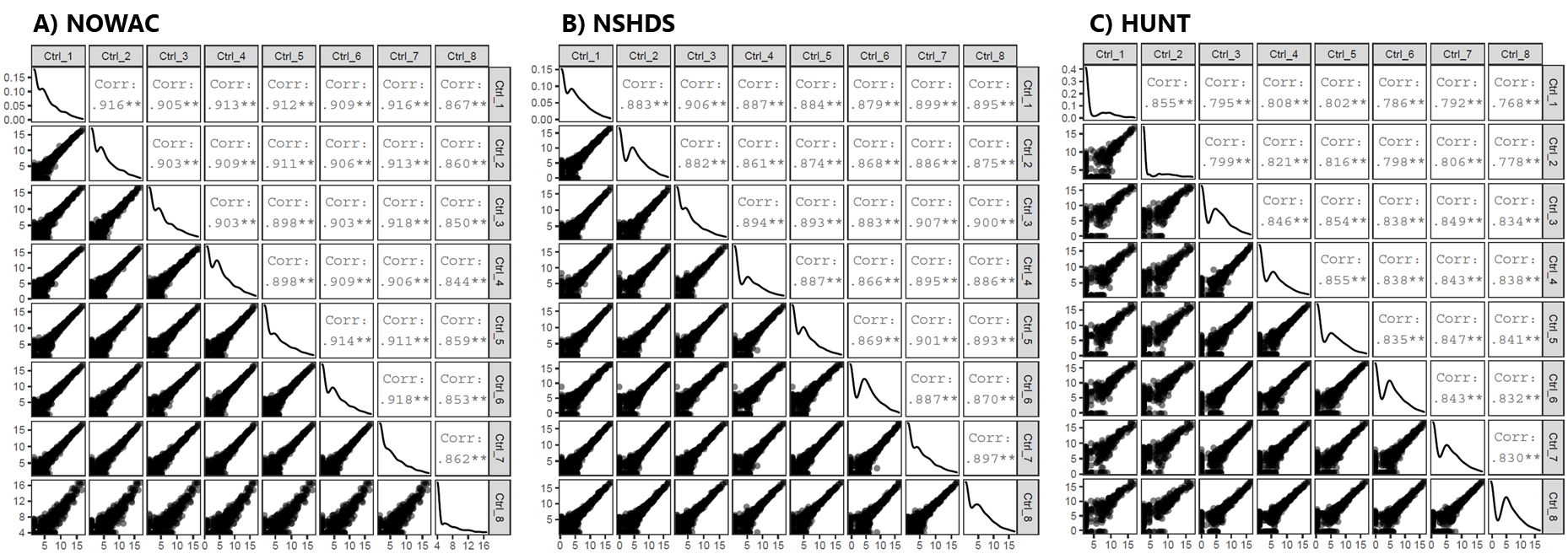


**Figure S4**: For the preprocessed data in A) NOWAC, B) NSHDS, C) HUNT, correlation of reads for those miRNAs that were detected >1 read per million for aliquots of an in-house pooled reference specimen prepared and analysed in batches together with specimens from A) NOWAC (n=8 aliqouts), B) NSHDS (n=8), C) HUNT (n=8).


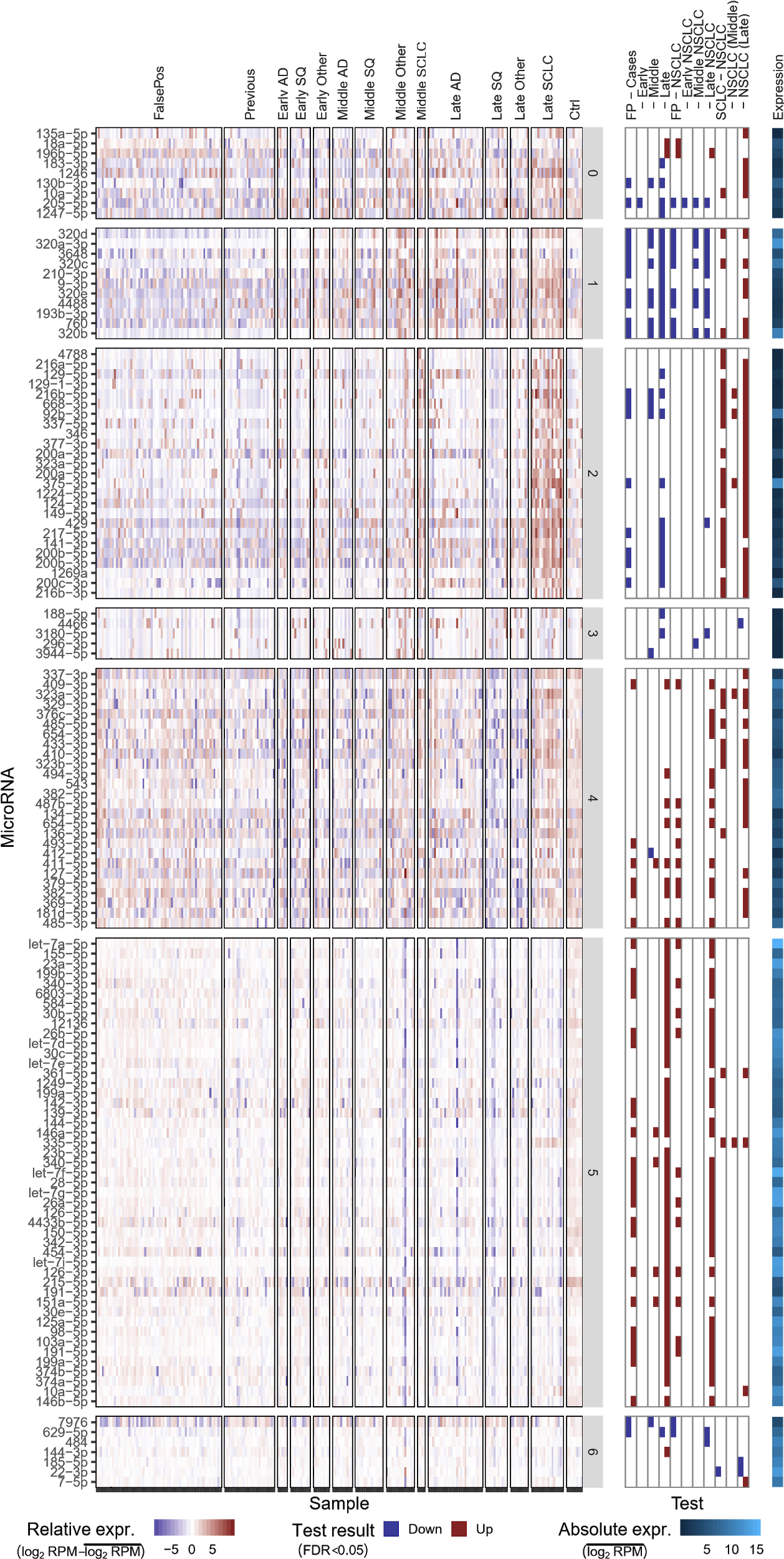


**Figure S5**: Heatmap displaying the relative expression of miRNAs in NLCB, the direction of differential expression and the absolute expression of the same miRNAs in order of appearance from left to right. The miRNAs in six clusters are plotted in the vertical direction of the plot.


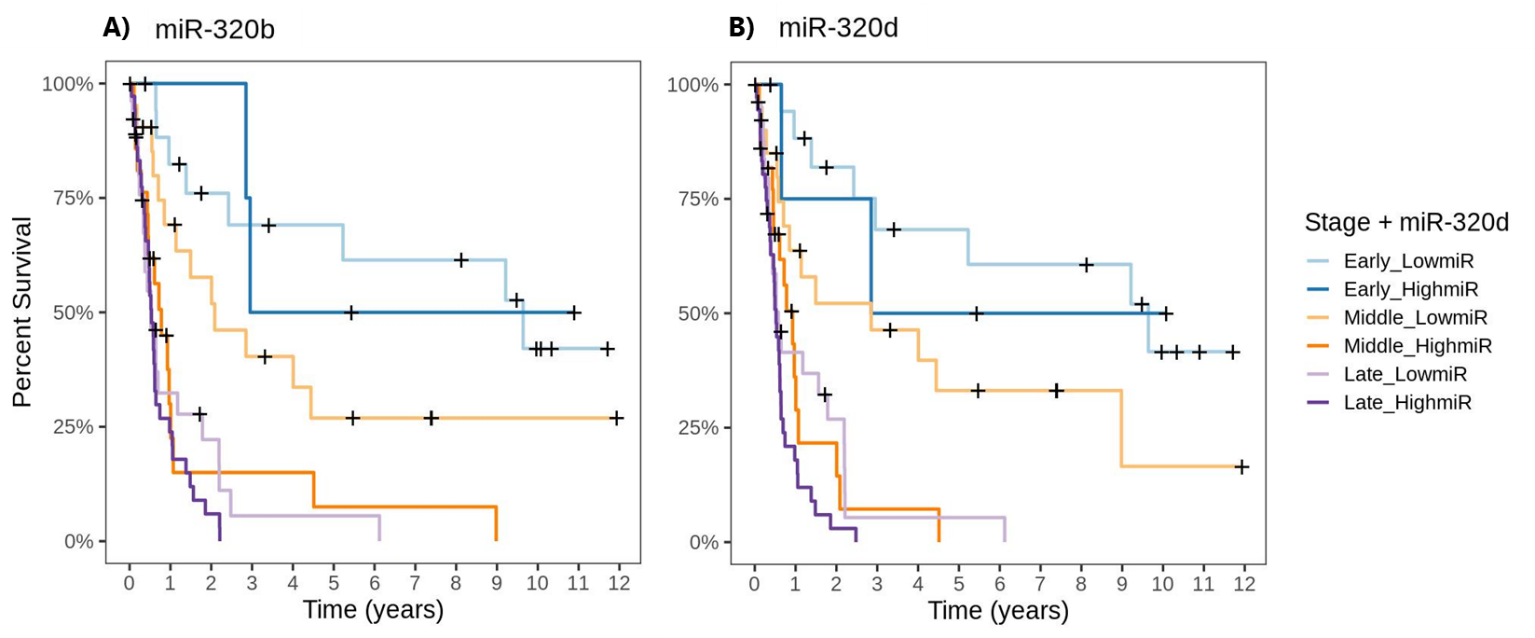


**Figure S6**: High expression of (A) miR-320b and (B) miR-320d was associated with lower survival in NLCB (*P*=0.02 and *P*=0.03, respectively). The survival curves are grouped by early (blue), middle (orange), and late (purple) stage cases, subdivided by low (“LowmiR”, light colors) or high (“HighmiR”, dark colors) expression of the respective miRNA. Low and high was defined by having, respectively, RPM expression below or above the median RPM of the respective miRNA. Cox models with lung cancer death as endpoint were used to assess significance; *P*-values were Bonferroni adjusted for multiple testing of the nine candidate miRNAs.

**
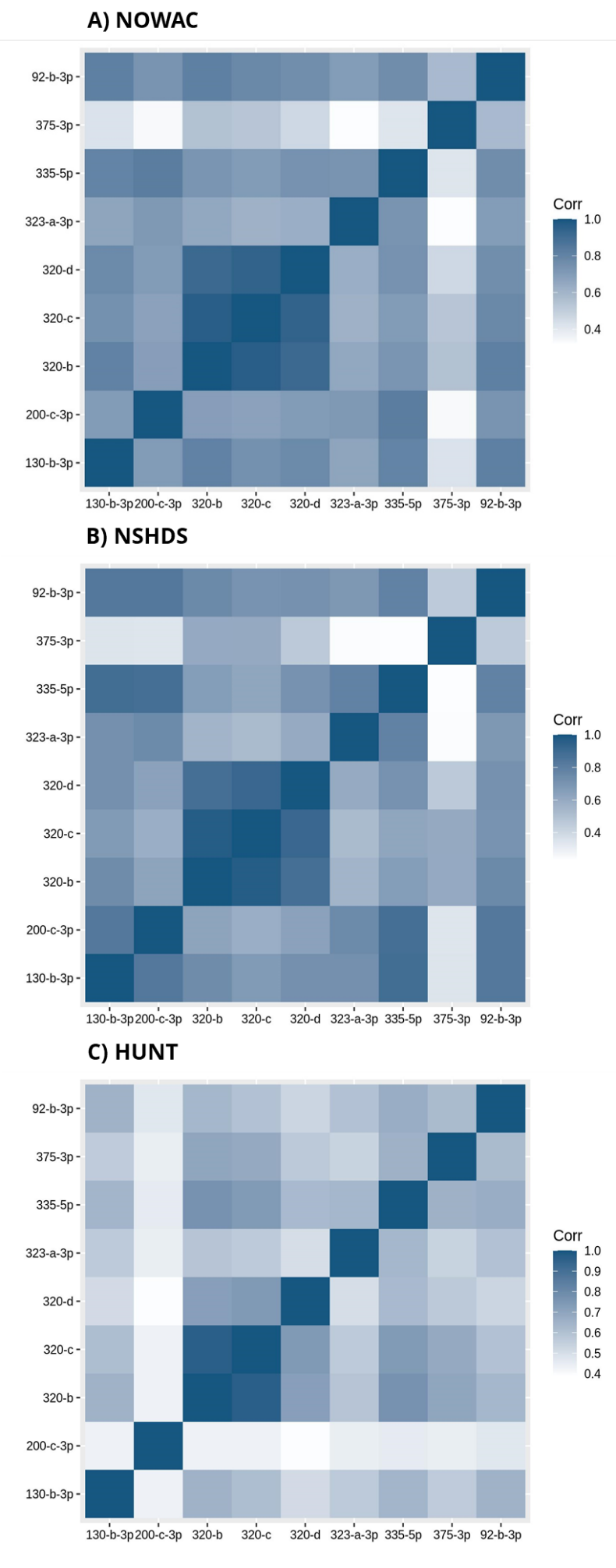
**

**Figure S7**: Heatmap of Pearson’s correlation coefficients for log2 read per million values for candidate miRNAs in the three pre-diagnostic studies (NOWAC, NSHDS, HUNT) combined.


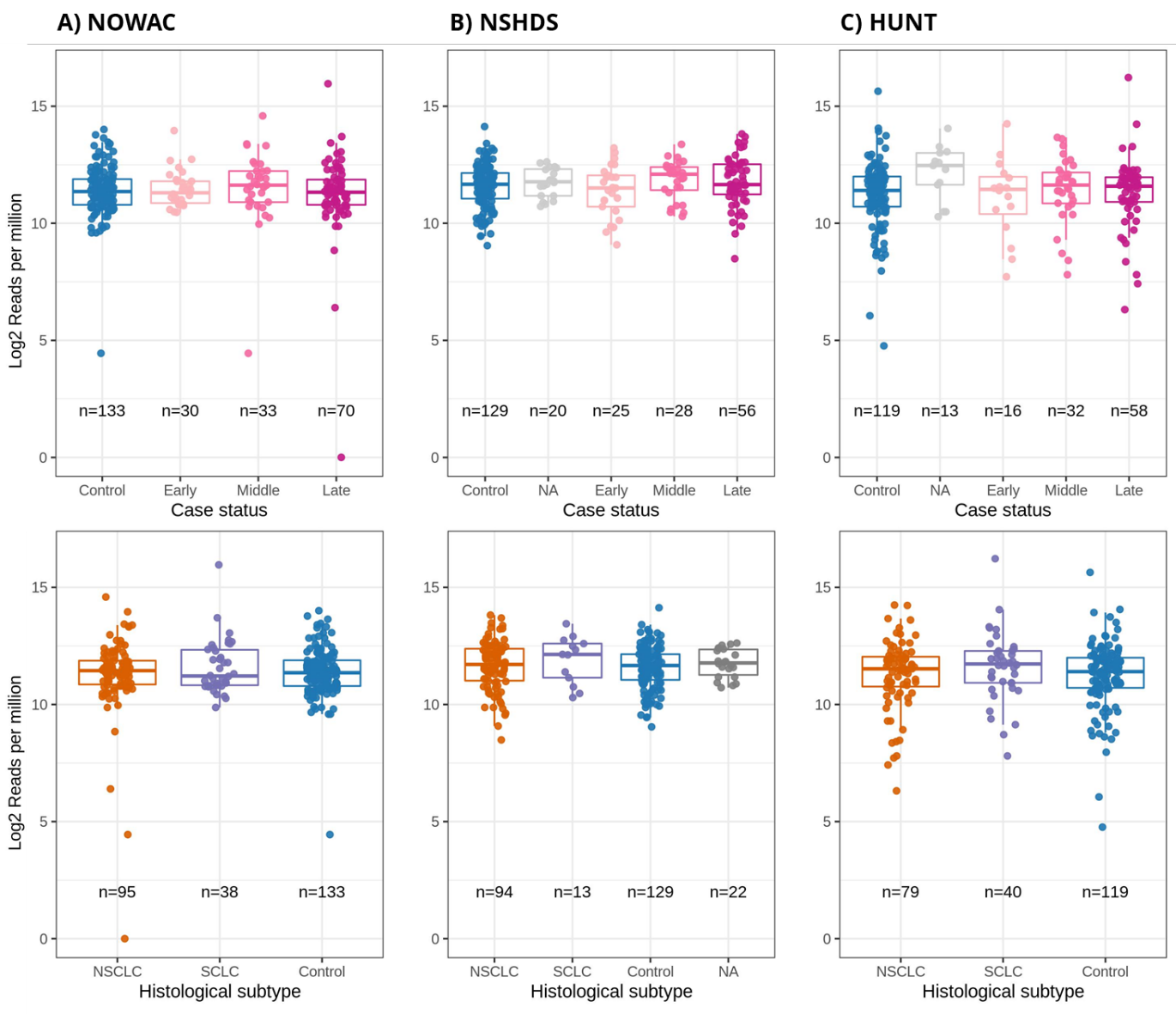


**Figure S8**: The distribution of log2 reads per million values for one selected candidate miRNA of interest, miR-320c, in controls and LC stage groups in upper panels, and histological subtypes in the lower panels for specimens from the A) NOWAC, B) NSHDS and C) HUNT studies.


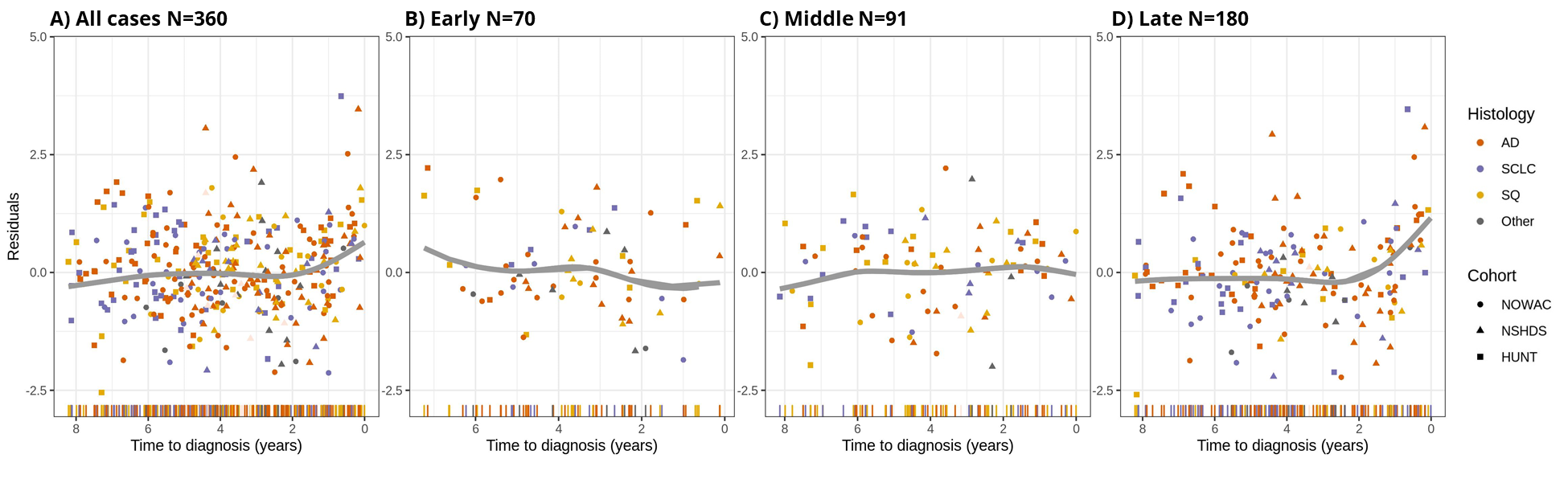


**Figure S9**: Representation of trends for miR-320d for LC cases in the pre-diagnostic specimens (NOWAC, NSHDS, HUNT) according to the number of days in the interval between time of specimen collection and time of diagnosis for A) all LC, B) early-stage LC, C) middle-stage LC, and D) late-stage LC. Specimens are colored according to histological subtypes (AD= adenocarcinoma LC, SCLC= small cell LC, SQ= squamous cell carcinoma LC, Other= other histological subtypes of LC). The values represent residuals from mixed models including log2 read per million values and matching factors (age, sex, smoking status) in addition to a random effect for the three studies included in our prospective study sample, to account for study-specific effects. Predicted values for splines with three knots for days prior to diagnosis in a generalized additive model are indicated.

**
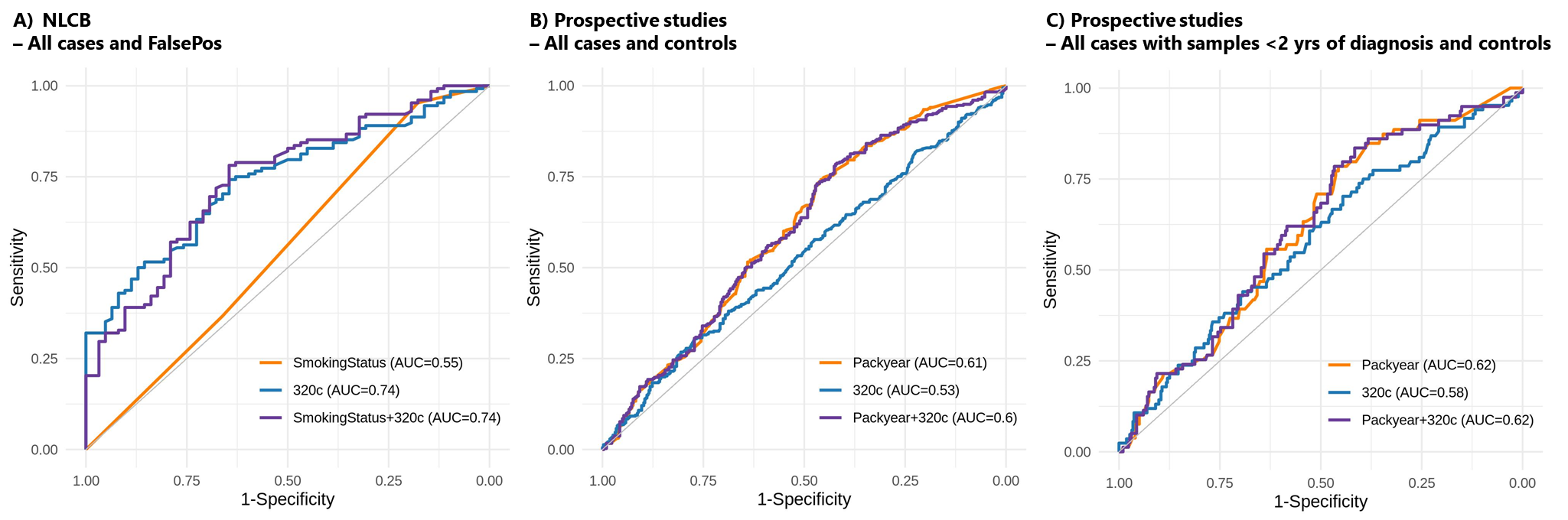
**

**Figure S10**: A) Case-FalsePos discrimination for specimens in NLCB (n=128 cases, 62 FalsePos), B) case-control discrimination in the prospective study sample (NOWAC, NSHDS, HUNT, n=373 cases, 375 controls), and C) case-control discrimination in the pre-diagnostic specimens collected from cases within two years of LC diagnosis (n=84 cases, 375 controls). Separate ROC curves are displayed for models including smoking status for NLCB or pack-years for prospective studies only, miR-320c expression only, and both the smoking variable and miR-320c expression.

**
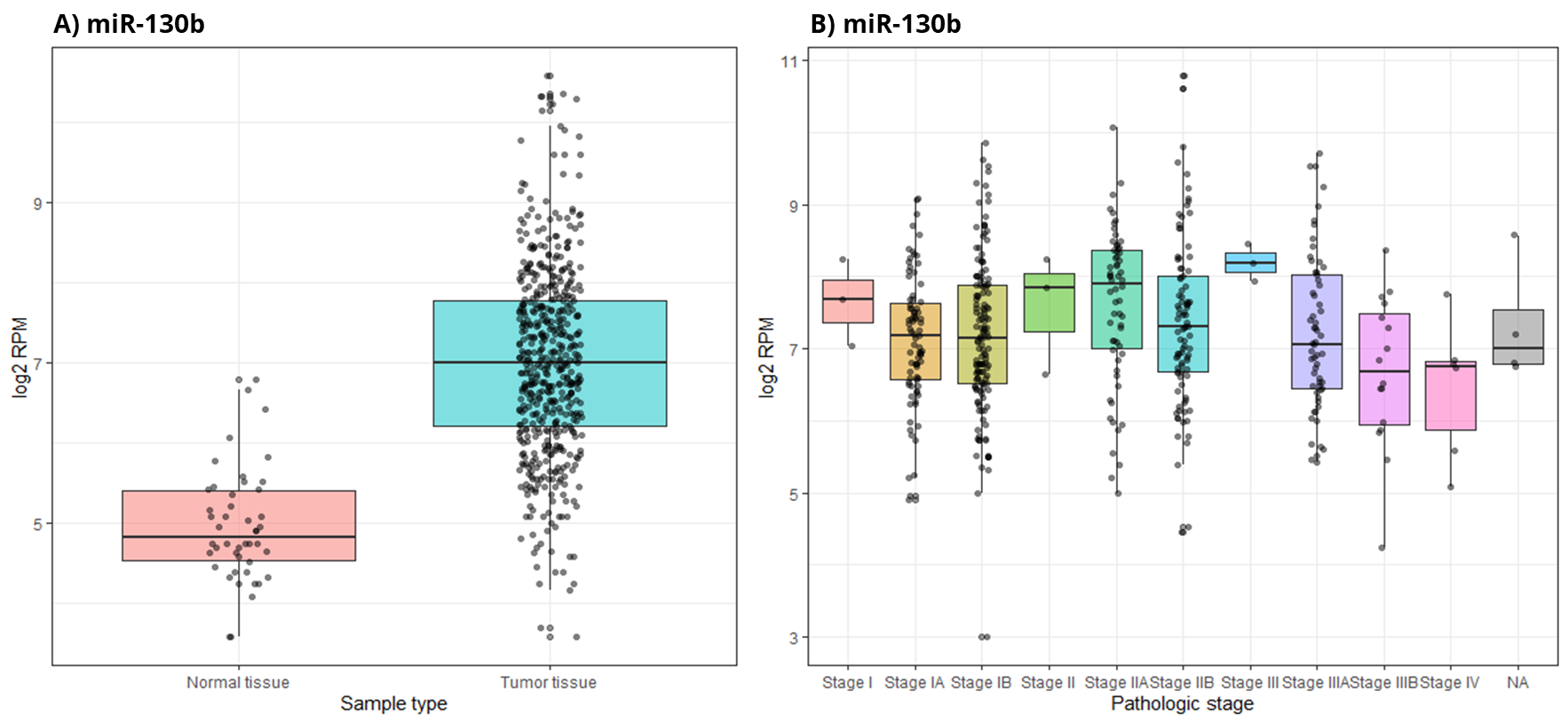
**

**Figure S11:** A) Expression of miR-130b in normal (N=46) and adenocarcinoma tumor tissue (N=519) in the TCGA dataset, B) Expression of miR-130b in squamous carcinoma tumor tissue (N=478) displayed for all pathological stages.

**Table S1:** The definition of cancer stage categories based on information either from the Cancer Registry of Norway (NOWAC and HUNT) or medical records (NLCB and NSHDS).

| **Cancer stage** | **NOWAC, HUNT** | **NLCB, NSHDS** |
| --- | --- | --- |
|  | **Cancer Registry - metastasis categories*** | **Medical records - M in TNM** |
| Early-stage | 0 | Stage I-II |
| Middle-stage | 1, 5, 6 | Stage III |
| Late-stage | 2, 3, 4, 7 | Stage IV |
| Unknown | 9 | Unknown |

*The coding of the categories in the registry was the following: 0 = No metastasis, 1 = Metastasis to regional lymph nodes, 2 = Metastasis to distant lymph nodes, 3 = Metastasis to organ in the same part of the body as the primary tumor, 4 = Metastasis to organ in another part of the body than the primary tumor, 5 = Microscopic growth into neighboring tissue, 6 = Macroscopic growth into neighboring tissue, 7 = Metastasis found, but uncertain where primary tumor is located, 8 = Microscopically infiltrating tumor, 9 = Unknown metastasis. None were coded 8

**Table S2**: The total number of differentially expressed miRNAs (considering an FDR *P*-value threshold), the maximum log FC, and minimum FDR-adjusted *P*-value in the different group tests considered.

| **Group test** | **Significant miRNAs** | **Maximum log FC** | **Minimum adjusted *P*-value** |
| --- | --- | --- | --- |
| All cases *vs* FalsePos | 46 | 2.24 | 9.06E-10 |
| Early-stage *vs* FalsePos | 1 | 2.89 | 2.29E-02 |
| Middle-stage *vs* FalsePos | 13 | 2.75 | 2.74E-07 |
| Late-stage *vs* FalsePos | 70 | 2.50 | 1.61E-09 |
| Early-stage *vs* middle-stage | 0 | 1.97 | 8.60E-01 |
| Early-stage *vs* late-stage | 0 | 2.06 | 4.20E-01 |
| Middle-late *vs* late-stage | 0 | 1.73 | 4.82E-01 |
| SCLC *vs* FalsePos | 65 | 4.43 | 9.63E-16 |
| NSCLC *vs* FalsePos | 16 | 2.47 | 6.55E-06 |
| SCLC *vs* NSCLC | 38 | 4.33 | 5.07E-16 |

**Table S3**: Model summaries for the nine candidate miRNAs in NLCB using R limma analyses. Presented are log2 fold change (logFC) values for the difference in expression between FalsePos and all LC cases (n=190), adjusted *P*-values, crude *P*-values, and average expression (AveExpr) for each miRNA. Models were adjusted for age, sex and lane on sequencing chip.

|  |  | **All cases *vs* FalsePos** | | | **Late-stage cases *vs* FalsePos** | | | **SCLC cases *vs* FalsePos** | | |
| --- | --- | --- | --- | --- | --- | --- | --- | --- | --- | --- |
| **miRs** | AveExpr | logFC | adj. *P*-value | *P*-value | logFC | adj. *P*-value | *P*-value | logFC | adj. *P*-value | *P*-value |
| miR-320d | 7.79 | 2.04 | **1.02E-10** | **1.41E-13** | 2.26 | **2.74E-10** | **3.79E-13** | 3.82 | **8.80E-12** | **3.65E-14** |
| miR-320c | 9.31 | 1.31 | **1.35E-06** | **3.74E-09** | 1.51 | **6.94E-07** | **1.92E-09** | 2.65 | **1.17E-08** | **8.13E-11** |
| miR-320b | 10.26 | 1.06 | **1.42E-05** | **5.91E-08** | 1.22 | **3.86E-06** | **3.36E-08** | 2.10 | **3.90E-07** | **3.78E-09** |
| miR-92b-3p | 8.27 | 0.70 | **7.99E-04** | **7.01E-06** | 1.00 | **3.86E-06** | **2.76E-08** | 2.46 | **1.50E-13** | **4.14E-16** |
| miR-130b-3p | 6.23 | 1.06 | **4.47E-03** | **8.65E-05** | 1.30 | **6.24E-04** | **2.22E-05** | 2.36 | **1.03E-04** | **1.84E-06** |
| miR-200c-3p | 5.52 | 1.06 | **2.03E-02** | **8.85E-04** | 1.48 | **9.89E-04** | **4.51E-05** | 2.69 | **2.49E-04** | **4.82E-06** |
| miR-375-3p | 10.93 | 0.82 | **3.29E-02** | **1.75E-03** | 1.31 | **4.06E-04** | **1.23E-05** | 4.36 | **9.32E-15** | **1.29E-17** |
| miR-335-5p | 9.86 | 0.25 | 4.16E-01 | 7.61E-02 | 0.17 | 6.25E-01 | 2.97E-01 | 2.02 | **5.13E-11** | **2.84E-13** |
| miR-323a-3p | 5.61 | 0.44 | 6.30E-01 | 2.39E-01 | 0.60 | 4.62E-01 | 1.60E-01 | 3.88 | **3.46E-06** | **3.83E-08** |

**Table S4**: Model summaries for the nine candidate miRNAs in NLCB using R limma analyses. Model summaries are presented for models corresponding to those presented in Table S3, but were additionally adjusted for smoking status (never/former/current).

|  | **All cases *vs* FalsePos** | | | **Late-stage cases *vs* FalsePos** | | | **SCLC cases *vs* FalsePos** | | |
| --- | --- | --- | --- | --- | --- | --- | --- | --- | --- |
| **miRs** | logFC | adj. *P*-value | *P*-value | logFC | adj. *P*-value | *P*-value | logFC | adj. *P*-value | *P*-value |
| miR-320d | 2.12 | **1.50E-11** | **2.08E-14** | 2.33 | **4.11E-11** | **5.68E-14** | 3.89 | **6.49E-12** | **2.69E-14** |
| miR-320c | 1.35 | **5.57E-07** | **1.54E-09** | 1.55 | **2.49E-07** | **6.90E-10** | 2.66 | **1.57E-08** | **1.31E-10** |
| miR-320b | 1.09 | **8.38E-06** | **3.48E-08** | 1.25 | **2.06E-06** | **1.71E-08** | 2.10 | **6.96E-07** | **6.73E-09** |
| miR-92b-3p | 0.72 | **6.60E-04** | **5.47E-06** | 1.01 | **2.06E-06** | **1.62E-08** | 2.43 | **8.47E-13** | **2.34E-15** |
| miR-130b-3p | 1.06 | **4.50E-03** | **9.54E-05** | 1.30 | **5.85E-04** | **2.10E-05** | 2.32 | **2.11E-04** | **4.08E-06** |
| miR-200c-3p | 1.00 | **3.28E-02** | **1.82E-03** | 1.42 | **1.72E-03** | **8.79E-05** | 2.64 | **4.63E-04** | **9.61E-06** |
| miR-375-3p | 0.80 | **3.88E-02** | **2.39E-03** | 1.29 | **4.52E-04** | **1.56E-05** | 4.31 | **7.02E-14** | **9.71E-17** |
| miR-335-5p | 0.27 | 3.70E-01 | 6.46E-02 | 0.17 | 6.36E-01 | 2.93E-01 | 2.09 | **2.16E-11** | **1.19E-13** |
| miR-323a-3p | 0.42 | 6.36E-01 | 2.63E-01 | 0.60 | 4.51E-01 | 1.56E-01 | 3.70 | **2.03E-05** | **2.25E-07** |

**Table S5**: Model summaries for the nine candidate miRNAs in NLCB, and in NOWAC, NSHDS, and HUNT studies. Odds ratio (OR) values, their confidence intervals and *P*-values are presented for comparisons of LC cases (n=128) and FalsePos (n=62) in NLCB, for case-control comparisons in all pre-diagnostic specimens (n=360 cases, 375 controls; NOWAC, NSHDS and HUNT studies) and for case-control comparisons in pre-diagnostic specimens collected within two years of LC diagnosis for the cases (n=84 cases, 375 controls).

| **miR** | **NLCB ^a^** | | | **NOWAC+NSHDS+HUNT All ^b^** | | | **NOWAC+NSHDS+HUNT <2 years to diagnosis ^b^** | | |
| --- | --- | --- | --- | --- | --- | --- | --- | --- | --- |
|  | OR | 95% CI | *P*-value | OR | 95% CI | *P*-value | OR | 95% CI | *P*-value |
| miR-320d | 2.46 | (1.83 - 3.32) | **3.00E-09** | 1.08 | (0.95 - 1.24) | 0.23 | 1.25 | (1.01 - 1.54) | **0.04** |
| miR-320c | 2.51 | (1.77 - 3.54) | **1.86E-07** | 1.11 | (0.96 - 1.28) | 0.16 | 1.24 | (1.00 - 1.54) | 0.06 |
| miR-320b | 2.85 | (1.91 - 4.26) | **3.28E-07** | 1.13 | (0.97 - 1.31) | 0.11 | 1.21 | (0.96 - 1.52) | 0.10 |
| miR-92b-3p | 1.89 | (1.35 - 2.65) | **2.03E-04** | 0.97 | (0.84 - 1.12) | 0.68 | 1.07 | (0.84 - 1.37) | 0.58 |
| miR-130b-3p | 1.53 | (1.24 - 1.90) | **1.01E-04** | 1.06 | (0.92 - 1.23) | 0.42 | 1.00 | (0.81 - 1.25) | 0.98 |
| miR-200c-3p | 1.45 | (1.22 - 1.73) | **2.62E-05** | 1.01 | (0.93 - 1.10) | 0.81 | 1.08 | (0.93 - 1.26) | 0.30 |
| miR-375-3p | 1.33 | (1.10 - 1.61) | **2.71E-03** | 1.03 | (0.94 - 1.14) | 0.51 | 1.07 | (0.92 - 1.26) | 0.37 |
| miR-335-5p | 1.22 | (0.88 - 1.70) | 2.26E-01 | 0.93 | (0.80 - 1.07) | 0.29 | 0.90 | (0.73 - 1.12) | 0.35 |
| miR-323a-3p | 1.07 | (0.95 - 1.22) | 2.75E-01 | 0.99 | (0.91 - 1.08) | 0.87 | 0.98 | (0.85 - 1.12) | 0.72 |

**^a^** Logistic models were adjusted for age, sex and lane on sequencing chip;

**^b^** Mixed models were adjusted for age, sex, and included study as random effect.

**Table S6**: Model summaries for the nine candidate miRNAs in *late-stage cases* in NLCB, and in NOWAC, NSHDS, and HUNT studies. Odds ratio (OR) values, their confidence intervals and *P*-values are presented for comparisons of LC cases (n=63) and FalsePos (n=62) in NLCB, for case-control comparisons in all pre-diagnostic specimens (n=180 cases, 375 controls; NOWAC, NSHDS and HUNT studies) and for case-control comparisons in pre-diagnostic specimens collected within two years of LC diagnosis for the cases (n=24 cases, 375 controls).

| **miR** | **NLCB ^a^** | | | **NOWAC+NSHDS+HUNT All ^b^** | | | **NOWAC+NSHDS+HUNT <2 years to diagnosis ^b^** | | |
| --- | --- | --- | --- | --- | --- | --- | --- | --- | --- |
|  | OR | 95% CI | *P*-value | OR | 95% CI | *P*-value | OR | 95% CI | *P*-value |
| miR-320d | 2.75 | (1.89 - 4.01) | **1.36E-07** | 1.11 | (0.94 - 1.30) | 0.22 | 1.38 | (0.94 - 2.03) | 0.10 |
| miR-320c | 3.39 | (2.08 - 5.51) | **9.22E-07** | 1.11 | (0.93 - 1.31) | 0.25 | 1.45 | (0.97 - 2.15) | 0.07 |
| miR-320b | 3.66 | (2.19 - 6.15) | **8.54E-07** | 1.15 | (0.96 - 1.37) | 0.12 | 1.38 | (0.93 - 2.05) | 0.10 |
| miR-92b-3p | 3.09 | (1.84 - 5.18) | **1.94E-05** | 1.01 | (0.84 - 1.21) | 0.93 | 0.99 | (0.67 - 1.46) | 0.96 |
| miR-130b-3p | 1.79 | (1.30 - 2.47) | **3.49E-04** | 1.06 | (0.89 - 1.27) | 0.53 | 0.77 | (0.56 - 1.04) | 0.09 |
| miR-200c-3p | 1.71 | (1.33 - 2.19) | **2.34E-05** | 1.01 | (0.91 - 1.13) | 0.80 | 1.11 | (0.86 - 1.44) | 0.41 |
| miR-375-3p | 1.53 | (1.22 - 1.92) | **2.62E-04** | 1.11 | (0.99 - 1.25) | 0.09 | 1.27 | (0.91 - 1.76) | 0.16 |
| miR-335-5p | 1.36 | (0.92 - 2.02) | 1.26E-01 | 0.88 | (0.74 - 1.05) | 0.15 | 0.79 | (0.58 - 1.09) | 0.15 |
| miR-323a-3p | 1.13 | (0.97 - 1.32) | 1.12E-01 | 1.00 | (0.90 - 1.11) | 0.98 | 0.84 | (0.69 - 1.03) | 0.10 |

**^a^** Logistic models were adjusted for age, sex and lane on sequencing chip;

**^b^** Mixed models were adjusted for age, sex, and included study as random effect.

| **miR** | **NLCB ^a^** | | | | | **NOWAC+NSHDS+HUNT All ^b^** | | | | | **NOWAC+NSHDS+HUNT <2 years to diagnosis ^b^** | | | | | |
| --- | --- | --- | --- | --- | --- | --- | --- | --- | --- | --- | --- | --- | --- | --- | --- | --- |
|  | OR | | 95% CI | | *P*-value | OR | | 95% CI | | *P*-value | OR | | 95% CI | | *P*-value | |
| miR-320d | 1.47E+04 | (0.02 - 1.45E+10) **^c^** | | 1.73E-01 | | 1.11 | (0.89 - 1.38) | | 0.36 | | 1.56 | (0.93 - 2.61) | | 0.09 | |  |
| miR-320c | 6.57E+04 | (0.12 - 3.60E+10) **^c^** | | 9.99E-02 | | 1.04 | (0.81 - 1.34) | | 0.77 | | 1.44 | (0.84 - 2.46) | | 0.18 | |  |
| miR-320b | 169.40 | (5.87 - 4.89E+03) **^c^** | | **2.78E-03** | | 1.08 | (0.83 - 1.40) | | 0.57 | | 1.46 | (0.88 - 2.44) | | 0.14 | |  |
| miR-92b-3p | 122.32 | (4.90 - 3.05E+03) | | **3.41E-03** | | 0.99 | (0.79 - 1.24) | | 0.95 | | 2.03 | (0.95 - 4.31) | | 0.07 | |  |
| miR-130b-3p | 17.88 | (3.91 - 81.85) | | **2.02E-04** | | 1.28 | (1.00 - 1.63) | | 0.05 | | 1.56 | (0.82 - 2.96) | | 0.18 | |  |
| miR-200c-3p | 245.04 | (6.22 - 9.65E+03) | | **3.33E-03** | | 0.93 | (0.80 - 1.08) | | 0.36 | | 1.20 | (0.78 - 1.85) | | 0.41 | |  |
| miR-375-3p | 9.68 | (2.18 - 43.00) | | **2.85E-03** | | 1.09 | (0.92 - 1.28) | | 0.31 | | 1.14 | (0.73 - 1.76) | | 0.57 | |  |
| miR-335-5p | 27.60 | (4.95 - 154.06) | | **1.55E-04** | | 0.90 | (0.70 - 1.17) | | 0.44 | | 1.07 | (0.57 - 2.00) | | 0.84 | |  |
| miR-323a-3p | 3.58 | (1.91 - 6.70) | | **6.64E-05** | | 0.96 | (0.83 - 1.12) | | 0.61 | | 0.93 | (0.66 - 1.31) | | 0.68 | |  |

**Table S7**: Model summaries of models for the nine candidate miRNAs in *SCLC cases* in NLCB and in NOWAC, NSHDS, and HUNT studies. Odds ratio (OR) values, their confidence intervals and *P*-values are presented for comparisons of LC cases (n=20) and FalsePos (n=62) in NLCB, for case-control comparisons in all pre-diagnostic specimens (n=91 cases, 375 controls; NOWAC, NSHDS and HUNT studies) and for case-control comparisons in pre-diagnostic specimens collected within two years prior to diagnosis for the cases (n=11 cases, 375 controls).

**^a^** Logistic models were adjusted for age, sex and lane on sequencing chip;

**^b^** Mixed models were adjusted for age, sex, and included study as random effect.

^c^ Models estimates inflated due to the approximate separation of the two groups (see Figure 1K).

| **miR** | **NLCB ^a^** | | | **NOWAC+NSHDS+HUNT All ^b^** | | | **NOWAC+NSHDS+HUNT <2 years to diagnosis ^b^** | | |
| --- | --- | --- | --- | --- | --- | --- | --- | --- | --- |
|  | OR | 95% CI | *P*-value | OR | 95% CI | *P*-value | OR | 95% CI | *P*-value |
| miR-320d | 2.29 | (1.69 - 3.12) | **1.25E-07** | 1.05 | (0.91 - 1.22) | 0.48 | 1.18 | (0.84 - 1.65) | 0.33 |
| miR-320c | 2.22 | (1.55 - 3.17) | **1.24E-05** | 1.10 | (0.94 - 1.28) | 0.24 | 1.41 | (1.00 - 1.99) | **0.05** |
| miR-320b | 2.46 | (1.62 - 3.73) | **2.30E-05** | 1.12 | (0.95 - 1.32) | 0.17 | 1.34 | (0.95 - 1.89) | 0.10 |
| miR-92b-3p | 1.54 | (1.06 - 2.23) | **2.39E-02** | 0.96 | (0.81 - 1.13) | 0.61 | 0.85 | (0.62 - 1.16) | 0.32 |
| miR-130b-3p | 1.41 | (1.13 - 1.75) | **2.21E-03** | 1.00 | (0.86 - 1.17) | 0.98 | 0.76 | (0.58 - 1.01) | 0.06 |
| miR-200c-3p | 1.34 | (1.12 - 1.60) | **1.13E-03** | 1.08 | (0.97 - 1.20) | 0.14 | 1.01 | (0.81 - 1.24) | 0.96 |
| miR-375-3p | 1.09 | (0.86 - 1.38) | 4.85E-01 | 1.00 | (0.90 - 1.11) | 0.98 | 1.13 | (0.87 - 1.47) | 0.36 |
| miR-335-5p | 0.75 | (0.48 - 1.18) | 2.15E-01 | 0.97 | (0.83 - 1.13) | 0.66 | 0.73 | (0.55 - 0.97) | **0.03** |
| miR-323a-3p | 0.95 | (0.82 - 1.11) | 5.32E-01 | 1.03 | (0.94 - 1.13) | 0.56 | 0.88 | (0.73 - 1.06) | 0.18 |

**Table S8**: Model summaries of models for the nine candidate miRNAs in *NSCLC cases* in NLCB and in NOWAC, NSHDS, and HUNT studies. Odds ratio (OR) values, their confidence intervals and *P*-values are presented for comparisons of LC cases (n=108) and FalsePos (n=62) in NLCB, for case-control comparisons in all pre-diagnostic specimens (n=269 cases, 375 controls; NOWAC, NSHDS and HUNT studies) and for case-control comparisons in pre-diagnostic specimens collected within two years prior to diagnosis for the cases (n=31 cases, 375 controls).

**^a^** Logistic models were adjusted for age, sex and lane on sequencing chip;

**^b^** Mixed models were adjusted for age, sex, and included study as random effect.

**Table S9**: Model summary for survival model for expression of miR-320c (scaled) in NLCB (LC cases, n=128). Hazard ratio (HR) values, their confidence intervals and *P*-values are presented.

| **Variable** | **HR** | **95% CI** | | ***P*-value** |
| --- | --- | --- | --- | --- |
| miR-320c | 1.54 | 1.24 | 1.91 | 9.09E-05 |
| Age | 1.51 | 1.19 | 1.91 | 5.90E-04 |
| Stage Middle | 4.24 | 1.91 | 9.42 | 3.86E-04 |
| Stage Late | 15.88 | 6.84 | 36.87 | 1.25E-10 |
| Sex Male | 1.57 | 1.01 | 2.43 | 4.32E-02 |
| Smoking Former | 2.14 | 0.81 | 5.65 | 1.23E-01 |
| Smoking Current | 3.54 | 1.23 | 10.22 | 1.94E-02 |
| Hist SCLC | 0.56 | 0.30 | 1.07 | 8.11E-02 |

**Table S10**: Model summaries for the nine candidate miRNAs in generalized additive models **^a^** allowing for non-linear trends in miRNA expression levels across time between blood specimen sampling and time of diagnosis in all cases in NOWAC, NSHDS, and HUNT (n=360).

| **miR** | **Sum of Squares** | **F-value** | ***P*-value** |
| --- | --- | --- | --- |
| miR-320d | 5.55 | 5.14 | **0.02** |
| miR-320c | 3.85 | 4.99 | **0.03** |
| miR-320b | 1.97 | 2.98 | 0.08 |
| miR-335-5p | 2.16 | 2.78 | 0.10 |
| miR-323a-3p | 2.12 | 0.85 | 0.36 |
| miR-92b-3p | 0.87 | 0.85 | 0.36 |
| miR-200c-3p | 0.83 | 0.41 | 0.52 |
| miR-130b-3p | 0.08 | 0.08 | 0.78 |
| miR-375-3p | 0.03 | 0.02 | 0.90 |

**^a^** Mixed models were adjusted for age, sex, and smoking status in addition to study as random effect, and residuals from models were extracted. A generalized additive model was then fitted for the residuals and trends were indicated using splines with three degrees of freedom.

**Table S11**: Model summaries for the nine candidate miRNAs in generalized additive models **^a^** allowing for non-linear trends in miRNA expression levels across time between blood specimen sampling and time of diagnosis in *late-stage cases* in NOWAC, NSHDS, and HUNT (n=180).

| **miR** | **Sum of Squares** | **F-value** | ***P*-value** |
| --- | --- | --- | --- |
| miR-320d | 7.94 | 8.21 | **4.7E-03** |
| miR-320c | 3.51 | 4.05 | 0.05 |
| miR-320b | 2.85 | 3.66 | 0.06 |
| miR-375-3p | 7.56 | 3.37 | 0.07 |
| miR-92b-3p | 3.33 | 2.76 | 0.10 |
| miR-200c-3p | 2.75 | 1.33 | 0.25 |
| miR-323a-3p | 0.62 | 0.29 | 0.59 |
| miR-335-5p | 0.10 | 0.13 | 0.72 |
| miR-130b-3p | 0.01 | 0.00 | 0.95 |

**^a^** Mixed models were adjusted for age, sex, and smoking status in addition to study as random effect, and residuals from models were extracted. A generalized additive model was then fitted for the residuals and trends were indicated using splines with three degrees of freedom.

**Table S12**: Model summaries for the nine candidate miRNAs in generalized additive models **^a^** allowing for non-linear trends in miRNA expression levels across time between blood specimen sampling and time of diagnosis in *SCLC cases* in NOWAC, NSHDS, and HUNT (n=91).

| **miR** | **Sum of Squares** | **F-value** | ***P*-value** |
| --- | --- | --- | --- |
| miR-320c | 3.31 | 4.26 | **0.04** |
| miR-92b-3p | 4.71 | 4.00 | 0.05 |
| miR-320d | 4.10 | 3.48 | 0.07 |
| miR-320b | 1.44 | 2.42 | 0.12 |
| miR-335-5p | 1.57 | 2.33 | 0.13 |
| miR-130b-3p | 0.91 | 1.33 | 0.25 |
| miR-200c-3p | 2.96 | 1.01 | 0.32 |
| miR-323a-3p | 2.23 | 0.84 | 0.36 |
| miR-375-3p | 1.17 | 0.45 | 0.51 |

**^a^** Mixed models were adjusted for age, sex, and smoking status in addition to study as random effect, and residuals from models were extracted. A generalized additive model was then fitted for the residuals and trends were indicated using splines with three degrees of freedom.

**Table S13**: Model summaries for the nine candidate miRNAs in generalized additive models **^a^** allowing for non-linear trends in miRNA expression levels across time between blood specimen sampling and time of diagnosis in *NSCLC cases* in NOWAC, NSHDS, and HUNT (n=269).

| **miR** | **Sum of Squares** | **F-value** | ***P*-value** |
| --- | --- | --- | --- |
| miR-320d | 1.80 | 1.74 | 0.19 |
| miR-320c | 1.12 | 1.47 | 0.23 |
| miR-320b | 0.80 | 1.18 | 0.28 |
| miR-335-5p | 0.81 | 1.02 | 0.31 |
| miR-375-3p | 1.38 | 0.73 | 0.39 |
| miR-130b-3p | 0.37 | 0.35 | 0.56 |
| miR-323a-3p | 0.52 | 0.22 | 0.64 |
| miR-92b-3p | 0.07 | 0.07 | 0.79 |
| miR-200c-3p | 0.01 | 0.00 | 0.94 |

**^a^** Mixed models were adjusted for age, sex, and smoking status in addition to study as random effect, and residuals from models were extracted. A generalized additive model was then fitted for the residuals and trends were indicated using splines with three degrees of freedom.

**Table S14**: Model summaries for the expression of candidate miRNAs in tumor (N =519) or normal (N = 46) lung tissue registered for adenocarcinoma in TCGA databases.

| **miR** | **OR** | **95% CI** | | ***P*-value** | **Mean expression Normal tissue** | **Mean expression Tumor tissue** |
| --- | --- | --- | --- | --- | --- | --- |
| miR-130b | 7.78 | 4.72 | 12.82 | **7.9E-16** | 34 | 182 |
| miR-323a | 2.08 | 1.60 | 2.71 | **4.5E-08** | 2 | 33 |
| miR-320b-2 | 2.06 | 1.58 | 2.68 | **9.4E-08** | 5 | 15 |
| miR-320b-1 | 2.15 | 1.61 | 2.86 | **1.9E-07** | 3 | 9 |
| miR-375 | 1.39 | 1.20 | 1.62 | **1.4E-05** | 35664 | 179704 |
| miR-92b | 1.60 | 1.29 | 1.98 | **1.6E-05** | 451 | 957 |
| miR-335 | 1.50 | 1.23 | 1.83 | **7.5E-05** | 454 | 607 |
| miR-200c | 1.54 | 1.24 | 1.92 | **7.9E-05** | 24031 | 51132 |
| miR-320c-2 | 2.44 | 1.56 | 3.82 | **9.5E-05** | 0 | 2 |
| miR-320d-1 | 1.50 | 1.09 | 2.07 | **1.3E-02** | 1 | 2 |
| miR-320d-2 | 1.51 | 1.08 | 2.11 | **1.7E-02** | 1 | 2 |
| miR-320c-1 | 0.97 | 0.72 | 1.31 | 8.5E-01 | 2 | 3 |

**Table S15**: Model summaries for the expression of candidate miRNAs in tumor (N = 478) or normal (N = 45) lung tissue registered for squamous cell carcinoma in TCGA databases.

| **miR** | **OR** | **95% CI** | | ***P*-value** | **Mean expression Normal tissue** | **Mean expression Tumor tissue** |
| --- | --- | --- | --- | --- | --- | --- |
| miR-375 | 0.39 | 0.31 | 0.50 | **7.6E-14** | 57456 | 19093 |
| miR-130b | 5.06 | 3.25 | 7.88 | **7.0E-13** | 60 | 202 |
| miR-320c-1 | 0.43 | 0.30 | 0.61 | **1.3E-06** | 4 | 2 |
| miR-335 | 0.54 | 0.41 | 0.71 | **1.0E-05** | 824 | 528 |
| miR-323a | 1.66 | 1.30 | 2.12 | **4.2E-05** | 5 | 29 |
| miR-92b | 0.61 | 0.47 | 0.78 | **1.3E-04** | 683 | 477 |
| miR-320d-2 | 0.59 | 0.43 | 0.81 | **8.7E-04** | 2 | 1 |
| miR-320d-1 | 0.89 | 0.65 | 1.21 | 4.6E-01 | 1 | 1 |
| miR-200c | 1.11 | 0.85 | 1.45 | 4.6E-01 | 32379 | 44080 |
| miR-320b-2 | 1.10 | 0.84 | 1.45 | 4.8E-01 | 11 | 17 |
| miR-320b-1 | 1.07 | 0.81 | 1.41 | 6.5E-01 | 7 | 10 |
| miR-320c-2 | 1.06 | 0.74 | 1.50 | 7.6E-01 | 1 | 1 |
